# Supplementary material for: Assault-related severe ocular chemical injury at a London ophthalmic referral hospital: a 3-year retrospective observational study
Source: BMJ Open. 2020 Oct 19;10(10):e038109. doi: 10.1136/bmjopen-2020-038109 (PMC7574930; doi:10.1136/bmjopen-2020-038109)
Supplement: Author's manuscript [file bmjopen-2020-038109.draft_revisions.pdf]

BMJ Open is committed to open peer review. As part of this commitment we make the peer review history of every article we publish publicly available.

When an article is published we post the peer reviewers' comments and the authors' responses online. We also post the versions of the paper that were used during peer review. These are the versions that the peer review comments apply to.

The versions of the paper that follow are the versions that were submitted during the peer review process. They are not the versions of record or the final published versions. They should not be cited or distributed as the published version of this manuscript.

BMJ Open is an open access journal and the full, final, typeset and author-corrected version of record of the manuscript is available on our site with no access controls, subscription charges or pay-per-view fees (http://bmjopen.bmj.com).

If you have any questions on BMJ Open's open peer review process please email info.bmjopen@bmj.com

BMJ Open

Assault with corrosive acid and alkali substances is now the leading cause of severe ocular chemical injury at a central London ophthalmic hospital

Journal:	*BMJ Open*
Manuscript ID	bmjopen-2020-038109
Article Type:	Original research
Date Submitted by the Author:	28-Feb-2020
Complete List of Authors:	Hoffman, Jeremy; Moorfields Eye Hospital NHS Foundation Trust, Accident and Emergency Department; University College London Institute of Ophthalmology,<br>Casswell, Edward; Moorfields Eye Hospital NHS Foundation Trust, Accident and Emergency Department; University College London Institute of Ophthalmology,<br>Shortt, Alex; Moorfields Eye Hospital NHS Foundation Trust; University College London Institute of Ophthalmology,
Keywords:	ACCIDENT & EMERGENCY MEDICINE, OPHTHALMOLOGY, Corneal and external diseases < OPHTHALMOLOGY, PUBLIC HEALTH

SCHOLARONE™
Manuscripts

I, the Submitting Author has the right to grant and does grant on behalf of all authors of the Work (as defined in the below author licence), an exclusive licence and/or a non-exclusive licence for contributions from authors who are: i) UK Crown employees; ii) where BMJ has agreed a CC-BY licence shall apply, and/or iii) in accordance with the terms applicable for US Federal Government officers or employees acting as part of their official duties; on a worldwide, perpetual, irrevocable, royalty-free basis to BMJ Publishing Group Ltd ("BMJ") its licensees and where the relevant Journal is co-owned by BMJ to the co-owners of the Journal, to publish the Work in this journal and any other BMJ products and to exploit all rights, as set out in our licence.

The Submitting Author accepts and understands that any supply made under these terms is made by BMJ to the Submitting Author unless you are acting as an employee on behalf of your employer or a postgraduate student of an affiliated institution which is paying any applicable article publishing charge ("APC") for Open Access articles. Where the Submitting Author wishes to make the Work available on an Open Access basis (and intends to pay the relevant APC), the terms of reuse of such Open Access shall be governed by a Creative Commons licence – details of these licences and which Creative Commons licence will apply to this Work are set out in our licence referred to above.

Other than as permitted in any relevant BMJ Author's Self Archiving Policies, I confirm this Work has not been accepted for publication elsewhere, is not being considered for publication elsewhere and does not duplicate material already published. I confirm all authors consent to publication of this Work and authorise the granting of this licence.

For peer review only

Assault with corrosive acid and alkali substances is now the leading cause of severe ocular chemical injury at a central London ophthalmic hospital

Authors: Jeremy John Hoffman (1, 2)*, Edward Joshua Casswell (1,2)*, Alex John Shortt (1, 2)

*- these authors contributed equally to the preparation of this manuscript

Affiliations:

(1) Moorfields Eye Hospital, London, UK

(2) NIHR Biomedical Research Centre, UCL Institute of Ophthalmology, London, UK

Conflict of interest:

All authors declare there is no conflict of interests.

Corresponding author:

Dr Jeremy Hoffman FRCOphth

UCL Institute of Ophthalmology

11-43 Bath Street

London, UK

EC1V 9EL

jeremy.hoffman@lshtm.ac.uk

Keywords:

Chemical injury, acid attack, corneal burns, ophthalmology, cornea

Word Count:

3115

For peer review only

ABSTRACT

Objectives:

To understand the incidence, causes, management and outcomes of severe ocular chemical injuries (SOCI) injuries at an urban tertiary referral centre in the UK.

Design:

Retrospective observational study.

Setting:

A London tertiary referral ophthalmic centre, Moorfields Eye Hospital.

Participants:

All cases of SOCI presenting between 1st September 2011 and 31st August 2014 were identified. The definition of SOCI was grade 3 or 4 on the Hughes-Roper-Hall classification system. We identified 25 cases (6 in 2011-12, 8 in 2012-13, 11 in 2013-14). Median age was 31.1 years. 23 cases (92%) were male.

Primary and secondary outcome measures:

The primary outcome was the proportion of cases of SOCI caused by assault, per year. Secondary outcome measures included the number of cases of SOCI, characteristics and mechanism of the injury, initial and long-term management, visual outcome and the need for surgical intervention.

Results:

Between 2011-12, 3/6 cases were due to assault (50%); between 2012-13, 7/8 were due to assault (87.5%); and between 2013-14, 6/11 were due to assault (54.4%). Assault was responsible for 16/25 (64%) cases overall, whilst 8/25 (32%) cases were work-related. The causative agent was known to be alkali in 15/25 (60%). 8/25 (32%) did not attend follow-up. The mean number of clock hours of limbal ischaemia was 5.24 (SD 2.97). 17/25 (68%) were Hughes-Roper-Hall grade 3. Surgical intervention occurred in 1/25. The final BCVA was worse than 6/12 in 11/25 (44%) and was CF in 3/25 (12%).

Conclusions:

Previous studies found that SOCI had a low incidence and that work-related accidents were the most common cause. Our study demonstrates an increasing incidence of SOCI which may be accounted for by a rise in assault using corrosive substances. Immediate or early irrigation was not always performed, a high number of patients did not attend regularly for follow-up and visual outcomes from these injuries are poor.

For peer review only

ARTICLE SUMMARY

Strengths and limitations of this study

- This is a large case series, spanning three years, from a tertiary ophthalmic hospital in London
- The primary outcome was the proportion of cases of SOCI caused by assault, per year
- The methodology differs to the previous national multicentre prospective (BOSU) study conducted between 2005-06 meaning comparisons should be made with caution

For peer review only

INTRODUCTION

Ocular chemical injury is an ophthalmic emergency and accounts for 11.5-22.1% of ophthalmic trauma.[1] Rapid emergency first-aid management with copious irrigation is crucial, followed by appropriate acute ophthalmic care.[2] Fortunately, most injuries are minor and accidental.[3] However, severe ocular chemical injuries (SOCI), although infrequent, carry a high risk of developing devastating, long-term sequelae and sight loss if not managed appropriately and promptly.[2]

There are several factors that influence the severity of the injury, including the agent itself (acid or alkali), its strength, length of contact with the eye, volume and concentration. SOCI usually results from strong acid or alkali agents.[4] Alkali injuries are often most severe, as the basic charged molecules can rapidly cross the corneal epithelium and stroma and enter the anterior chamber.[2]

Several grading systems for ocular chemical injuries exist. Hughes noted the relationship between initial presenting clinical signs and prognosis; this was subsequently amended by Ballen and then Roper-Hall.[5-7] Although more recent systems have been developed, the Hughes-Roper-Hall (H-R-H) classification system is easy to use and remember, maintaining its popularity particularly in the ophthalmic emergency department.[8] The H-R-H system divides the injury into four grades. Grade 1 cases are mild with corneal epithelial damage only and no limbal ischaemia with a clear cornea. Grade 2 demonstrates a hazy cornea but with iris details still visible and less than one-third (three clock hours) limbal ischaemia. Grade 3 involves between one-third and one-half limbal ischaemia (three to six clock hours), hazy cornea obscuring iris details and complete epithelial loss. Grade 4, the most severe, presents with an opaque cornea and greater than one-half limbal ischaemia. H-R-H Grades 3 and 4 are considered SOCI.[3] The most recent evolution of burns classification is the Dua classification which includes an assessment of the degree of conjunctival burn involvement.[9]

A surveillance study of SOCI conducted in the UK from December 2005 to November 2006 only identified 12 cases this year, with a minimum estimated incidence of 0.02 per 100,000.[3] They found that 8/12 (66.7%) patients with SOCI were working-age men and half (6/12) occurred at work. Assault accounted for 4/12 (33.3%) of injuries.[3] These findings are supported by previous studies investigating mild, as well as severe ocular chemical injuries.[1,4,10] A more recent study in the US showed similar demographics, although assault only accounted for 2 out of 640 cases (0.31%).[11]

For peer review only

In the UK, particularly in metropolitan centres such as London, there has been a rapid rise in chemical assault or so-called "acid" attacks.[12] This increase has received significant attention in the press with a number of high-profile attacks, with the incidence increasing by 65% in 2017 alone.[13] In London, the Metropolitan Police recorded an increase from 162 cases in 2012 to 454 in 2016; this number is higher than all the chemical assaults recorded in India that year.[12]

The aim of this present study was to investigate the number and causes of SOCI presentations per year over a three-year period at Moorfields Eye Hospital, a central London tertiary referral eye hospital with a 24-hour ophthalmic emergency department. In addition, this study aimed to describe the demographics, responsible agent, clinical presentation and management of SOCI cases.

For peer review only

MATERIALS AND METHODS

This study was a retrospective, observational study of patients attending Moorfields Eye Hospital Emergency Department with severe ocular chemical injuries. The study received approval from the Moorfields Eye Hospital Research on Anonymised Data (ROAD) Ethics Committee in February 2015 (Ref: ROAD 15/010) and the Moorfields Eye Hospital Clinical Audit Department (Ref: CA15/CED/11).. The study was compliant with the Declaration of Helsinki. Formal patient consent was deemed not necessary by the Moorfields ROAD Ethics Committee.

Severe ocular chemical injuries were defined as either grade 3 or 4 on the Hughes-Roper-Hall (H-R-H) classification system.[5,7] The H-R-H classification was chosen over the newer Dua classification to follow the same inclusion criteria as reported in the BOSU Study.[3] Furthermore, this classification is what is currently used and documented in our Emergency Department, with the treatment protocol dependent on the H-R-H grade. All electronic records of emergency department attendances between 1st September 2011 and 31st August 2014 were searched for the following coded diagnoses "Chemical Injury or Trauma" or "Chemical Trauma" who were coded as either being admitted or followed-up in clinic. All paper records of these attendances were then reviewed. Cases meeting the SOCI definition above were included in the study.

The primary outcome was the proportion of cases of SOCI caused by assault, per year. Secondary outcomes included the number of cases of SOCI, characteristics and mechanism of the injury, initial and long-term management, visual outcome and the need for surgical intervention.

Statistical analysis was performed using STATA 14 (StataCorp, College Station, TX). Multivariable logistic regression models (for categorical outcomes) and multivariable linear regression models (for continuous outcomes) were fitted to assess whether poor visual outcome (defined as final BSCA 0.30 logMAR or worse) were independently associated with demographics, presenting features, mechanism of injury and management after adjusting for potential confounding factors. Likelihood ratio tests were used to assess the strength of association of factors with visual outcome.

For peer review only

Patient and Public Involvement

Following the publication of our research in an open-access, peer reviewed journal, we plan on raising the awareness of the risks of chemical injury to the eye and measures to prevent this from happening, through public awareness campaigns. We would also seek to involve first-responders in disseminating the advice on the emergency treatment of ocular chemical injuries.

RESULTS

There were 242 attendances coded as either “Chemical Injury or Trauma” or “Chemical Trauma” over the study period. Complete clinical records were available and reviewed for 219 individuals, whilst the electronic records were available for 19 cases. There were three duplicate records and one record was unavailable or missing. The retrieval rate for the study was therefore 99.6%. Of the 238 notes reviewed, 25 cases met the inclusion criteria of SOCI.

Demographics and mode of injury

The median age at presentation was 31.1 years and 23/25 cases (92%) were male. Assault was responsible for 16/25 (64%) cases across the three years, whilst 8/25 (32%) cases were work-related. Injury with an alkali substance was reported in 15/25 (60%) and unknown in 6/25 (24%), Figure 1.

There were 6 cases of SOCI identified between 2011-12, of which 3/6 were due to assault (50%); 8 between 2012-13, 7/8 due to assault (87.5%); and 11 between 2013-14, 6/11 due to assault (54.4%). During the same period, there were 77,652 encounters in the emergency department in 2011-12; 81,899 in 2012-13; and 85,799 in 2013-14. This gives a presentation rate of SOCI at Moorfields of 0.773 cases per 10,000 encounters in 2011-12; 0.977 cases per 10,000 encounters in 2012-13; and 1.28 cases per 10,000 encounters in 2013-14. There was a mean year-on-year increase of 0.254 cases per 10,000 encounters. There was an approximate increase of 65.6% SOCI encounters from 2011-12 to 2013-14.

Clinical Presentation

At presentation, the visual acuity was 6/18 or worse in 15/25 (60%) of patients, with 3/25 (12%) of patients 6/60 or worse, Table 1. The mean number of clock hours of limbal ischaemia was 5.24 (SD 2.97). There were 17/25 (68%) cases of H-R-H Grade 3 and 8/25 (32%) cases of H-

For peer review only

R-H Grade 4. Time to irrigation data were available in 9/25 cases: irrigation was performed within one hour of the index event in only 5/9 (55%) of these cases, whilst the mean time to irrigation was 2.6 hours (range 0.5 – 15 hours; SD 4.70).

Management

The Initial management instigated in our Emergency Department is described in Figure 2. All patients received a topical antibiotic and steroid at presentation, 22/25 (88%) of whom received preservative-free formulations. Topical citrate and ascorbate were prescribed in 18/25 (72%) and 21/25 (84%) of cases respectively. Oral ascorbate and oral doxycycline were administered to 19/25 (76%) and 15/25 (60%) of patients respectively. The full recommended protocol of preservative free antibiotics and steroids; topical citrate and ascorbate; and oral ascorbate and doxycycline was followed in 5/25 (20%).

Long term management and outcome

Of the 25 patients with H-R-H grade 3 to 4 injuries, 10/25 (40%) failed to comply with standard follow up regimes in our department and were lost to follow-up at various stages of treatment. 2/10 patients did not attend for review beyond one week and were discharged, 6/10 did not attend reviews beyond one month and 2/10 failed to attend appointments between 3 and 12 months, despite being offered alternative appointments, as per hospital policy. The final BCVA (defined here as the later of either the last clinic appointment in the patient records prior to discharge, or one year following the index event) was less than 6/12 in 44% of cases. There were 3/25 (12%) of patients with vision at CF or worse at final follow-up, Table 1.

Long term complications included keratoconjunctivitis sicca (6/25), persistent epithelial defects (4/25), microbial keratitis (1/25), central pannus (2/25), central corneal scarring (8/25), entropion (1/25) and cataract (1/25), Table 1. Of note, there were no recorded cases of corneal melt, perforation, secondary glaucoma or patients requiring eye removal.

Surgical intervention was performed in 1/25 (4%), who underwent an amniotic membrane graft, a limbal stem cell graft and finally a penetrating keratoplasty. This same patient subsequently developed cataract and underwent uncomplicated phacoemulsification and intra-ocular lens insertion.

For peer review only

Multivariable logistic regression modelling showed no statistically significant associations between presenting features, mode of injury, agent or initial management and final visual acuity.

For peer review only

Patient	BCVA at presentation	BCVA at final follow-up	H-R-H Grade	Surgical intervention	Long-term complications	Lost to follow-up?
1	6/9	6/4	3	-		No
2	6/18	6/18	3	-	KS, CCS	No
3	6/24	6/6	3	-		No
4	6/6	6/9	3	-	CCS	No
5	6/12	6/5	3	-		DNA at 1/52
6	6/6	6/9	3	-	KS	No
7	6/24	Unknown	2	-		DNA at 1/52
8	6/9	6/12	4	-		DNA at 1/12
9	6/9	6/5	3	-		No
10	6/24	6/6	4	-	KS, CCS, CP	No
11	6/9	6/18	4	-		DNA at 1/12
12	6/18	6/6	3	-	CCS	No
13	6/12	6/12	3	-		DNA at 1/12
14	6/36	6/9	4	-		DNA at 1/12
15	6/24	6/12	4	-		DNA at 1/12
16	6/18	6/9	3	-	KS, CCS	No
17	CF	HM	4	-	PED,	No
18	6/18	6/12	3	-		No
19	4/60	3/60	3	-		DNA at 3/12
20	6/12	HM	3	-	KS, PED, CCS	DNA at 12/12
21	6/36	HM	4	-	KS, PED, MK, CCS, CP, Ent	No
22	6/36	6/6	3	-		DNA at 1/12
23	6/12	6/5	3	-		No
24	3/60	6/12	4	-		No
25	6/18	6/9	3	AMG, LSC, PK, Phaco	PED, CCS, Cat	No

Table 1: Best corrected visual acuity (BCVA) at presentation and at final follow-up, H-R-H grade, surgical intervention and corneal sequelae for all patients, and whether patients were lost to follow-up. (H-R-H, Hughes-Roper-Hall; DNA, Did Not Attend follow-up; AMG, amniotic membrane graft; LSC, limbal stem cell graft; PK, penetrating keratoplasty; Phaco, phacoemulsification and intra-ocular lens insertion; KS, keratoconjunctivitis sicca; CCS, central corneal scar; CP, central pannus; PED, persistent epithelial defect; MK, microbial keratitis; Ent, entropion)

For peer review only

DISCUSSION

The number of cases of SOCI presenting to Moorfields Eye Hospital increased year-on-year between 2011 and 2014, at a mean increase of 0.254 cases per 10,000 encounters per year. There was an increase of 65.6% from 2011/12 to 2013/14. The number of cases attributable to assault doubled from 3/6 (50%) to 7/8 (87.5%) between 2011/12 and 2012/13. In 2013/14 there were 6/11 cases of SOCI caused by assault. Overall across the three years, assault was responsible for 16/25 (64%) presentations.

These results contrast to those of the BOSU study from 2005-06.[3] The calculated incidence in 2005 was 0.02 per 100 000, based on the 12 patients reported over the course of the year and assuming a UK population of 60 million. We report that in 2013/14 there were 11 cases of SOCI seen within one tertiary eye unit in Central London. Assuming that Moorfields Eye Hospital serves half of the London population for ophthalmic services and a population of 8.79 million, we estimate that the incidence of SOCI is 0.25 per 100 000 in London. This is more than 10 times greater than the 2005-06 study. Furthermore, we have found that the proportion of injuries due to assault compared to work-related is 64% to 28% respectively; this contrasts to the BOSU results of 33% and 50% respectively. Demographic characteristics between the two studies are similar with SOCI being more common in young males, although we found a greater proportion of males (92% compared to 75%).

The authors of the BOSU study anticipated a maximum of 200-300 cases of SOCI annually in the UK, based on two previous studies.[3,4,10] If our results were extrapolated across the UK with a population of 65.6 million, we would expect approximately 164 case of SOCI annually. This is closer to Macdonald *et al's* anticipated level of cases. However, it must be emphasized that the methodology differs significantly between the two studies: one is a retrospective review of cases at a single tertiary centre whilst the other is a prospective survey conducted across the UK. This means that direct comparisons and any conclusions should be made with caution. It is also likely that the BOSU study may have under-reported the results by up to 25%.[3] With our study being conducted in a tertiary centre, our results may over-estimate the incidence. Furthermore, it is likely that chemical injuries due to assault are higher in cities, and possibly in London, compared to the rest of the UK. We chose to conduct this present study given anecdotal evidence of an increase in SOCI at our centre. As opposed to the prospective methodology used in BOSU surveillance studies, this enables a “snapshot” to gauge trends. We would therefore advocate further nationwide multicentre prospective studies to evaluate the true incidence of SOCI.

For peer review only

The reasons behind the rise in “acid-attacks” are likely multifactorial. In the past, acid attacks were previously related to robberies. Now, with tougher legislation against carrying knives, corrosive substances were seen as a replacement weapon that was harder to detect, with no legislation previously in the UK against carrying it. However, the Offensive Weapons Act 2019, closes this loophole and places restricitons on the sale of corrosive substances and makes it an offensive to have a corrosive substance in a public place. [14] Furthermore, a person found guilty is liable for a fine and/or imprisonment for up to 12 months. This is in addition to sentencing guidelines that classify corrosive substances as “highly dangerous weapons”, meaning longer custodial sentences for those found guilty of using a corrosive substance to threaten or assault others.[15] Many of these attacks are related to gangs, with men being twice as likely as women to be the victims.[12]

An interesting result from our study is that almost one third (8/25, 32%) of our patients were lost to follow-up as they did not attend (DNA) subsequent review appointments. This raises several observations. Firstly, this may affect the results of our study. For example, the final visual acuity may be better than we reported if patients with better vision were more likely to DNA. Secondly, this rate is considerably higher than that reported by previous studies of ophthalmic outpatient attendances in the UK where the reported rate is 12.5-12.9%.[16,17] This is likely to be due to demographics; DNA rates are typically higher in young men, those from deprived backgrounds and those living in urban areas.[18] This patient demographic is likely over-represented in our study population, compared to the general ophthalmic population. The proportion of patients assaulted was equal between those who attended all appointments and those who were lost to follow up due to DNA, suggesting that assault itself was not a predictor for patients who eventually DNA. This finding has important implications for outpatient management: patients attending with SOCI should receive additional counselling on the importance of their follow-up appointments and may benefit from additional reminders via telephone calls, emails or text messages.

The most important step to treating chemical injury and improving the outcome is immediate irrigation – “dilution is the solution”.[1,4,19] This is ideally done immediately at the scene following the exposure, continued by the paramedical team in the ambulance on the way to hospital and repeated again at presentation until the pH has normalised. This should be performed with normal saline where available, but if this is not available, clean water should be used. In our study, this was performed in 55% of patients within one hour of the injury, less than the 75% reported by MacDonald *et al.*[3] There is therefore a need to improve people’s awareness of the importance of irrigation. Given the shift in the mode of injury from workplace-related injury, for which there already exists robust health and safety legislation and

For peer review only

awareness, to assault-related, we suggest raising awareness amongst people at risk of falling victim to “acid-attacks” through appropriately targeted advertising campaigns.

The aim of early medical management is to reduce inflammation and promote corneal re-epithelialisation, and prevent acute complications such as infection or corneal melt.[2] Treatment should avoid the use of preserved medications, particularly those containing phosphates, as this can precipitate acute corneal calcification.[20,21] Acute treatment therefore involves preservative-free topical steroids and antibiotics to reduce inflammation and prevent infection. Topical citrate and ascorbate helps to prevent corneal melts.[22] Oral doxycycline can reduce the inflammation and the risk of melt.[2] Oral ascorbate 1g daily can also prevent collagen lysis, promote healing and prevent corneal melts.[2] The protocol at Moorfields Eye Hospital is that all patients with SOCI receive preservative-free steroid and antibiotic, topical ascorbate and citrate, oral ascorbate and doxycycline and cycloplegia. In our study, whilst all patients received topical steroid and antibiotics, 76% (19/25) received preservative-free formulation. This has improved since the BOSU study where only half (6/12) of patients received preservative-free formulations.[3] Topical citrate and oral doxycycline were prescribed in 48% of patients (12/25), compared to 25% (3/12) of patients in the BOSU study.[3]

Surgical intervention was low in our study, with only one patient (1/25, 4%) undergoing surgical treatment. This same patient underwent multiple forms of ocular surface reconstructive surgery. This low surgical rate of intervention seen in our study compares to 50% (6/12) of cases in the BOSU study, of which half of these had more than one surgical procedure.[3] Reasons for this are unclear, but it is possible that the high DNA rate may have resulted in a falsely low rate of surgical intervention, with patients being lost to follow up before surgical intervention was appropriate.

Final vision was reduced to worse than 6/12 in 44% of cases, which is marginally better compared to 50% reported by MacDonald et al.[3] The rate of very severe visual loss (worse than 6/60) was 16% in our study, comparable to 16.7% in the BOSU study.[3] This suggests that on the whole, visual prognosis from SOCI remains poor, with the potential for significant morbidity and ocular sequelae as a result of the injury.

This study suggests that there has been a recent increase in the incidence of severe ocular chemical injury in a central London tertiary eye hospital, with assault now responsible for between one half and just under two-thirds of cases. It is likely that SOCI is more common than has previously been reported, particularly in metropolitan areas. This increase may be a

For peer review only

result of the recent increase in “acid attacks”, although further work is necessary to establish causality. Regardless, there is a need to reverse the increase in “acid” attacks through stronger legislation to act as a deterrent. There was a lower than expected level of initial irrigation and so public awareness of how to manage chemical injury could be improved. Similarly, first responders and emergency department personnel should be reminded of the importance of copious irrigation and onward referral to a specialist ophthalmic unit. Ophthalmologists attending these cases should follow an evidenced-based protocol and be aware of the risk of patients not attending their follow-up appointments and plan accordingly. An updated nationwide surveillance study is warranted to enable direct comparisons to what was seen nearly 15 years ago.

Funding:

Jeremy Hoffman was supported by the National Institute for Health Research (NIHR) Biomedical Research Centre based at Moorfields Eye Hospital NHS Foundation Trust and UCL Institute of Ophthalmology. Alex Shortt was supported by a Wellcome Trust Intermediate Clinical Fellowship based at the UCL Institute of Ophthalmology and the UCL Institute of Immunity and Transplantation Ed Casswell was supported through a grant from the Friends of Moorfields Eye Hospital Charity. The views expressed are those of the author(s) and not necessarily those of the Wellcome Trust, NHS, the NIHR or the Department of Health. There was no specific funding for this research.

For peer review only

References

1 Wagoner MD. Chemical injuries of the eye: current concepts in pathophysiology and therapy. *Survey of Ophthalmology* 1997;**41**:275–313.

2 Tuft SJ, Shortt AJ. Surgical rehabilitation following severe ocular burns. *Eye (Lond)* 2009;**23**:1966–71. doi:10.1038/eye.2008.414

3 Macdonald ECA, Cauchi PA, Azuara-Blanco A, *et al.* Surveillance of severe chemical corneal injuries in the UK. *British Journal of Ophthalmology* 2009;**93**:1177–80. doi:10.1136/bjo.2008.154831

4 Kuckelkorn R, Schrage N, Keller G, *et al.* Emergency treatment of chemical and thermal eye burns. *Acta Ophthalmol Scand* 2002;**80**:4–10.

5 Hughes WF. Alkali burns of the eye; clinical and pathologic course. *Arch Ophthal* 1946;**36**:189–214.

6 Ballen PH. Treatment of chemical burns to the eye. *Eye Ear Nose Throat Mon* 1964;**43**:57–61.

7 Roper-Hall MJ. Thermal and chemical burns. *Trans Ophthalmol Soc U K* 1965;**85**:631–53.

8 Harun S, Srinivasan S, Hollingworth K, *et al.* Modification of classification of ocular chemical injuries. *British Journal of Ophthalmology* 2004;**88**:1353–4–authorreply1354–5. doi:10.1136/bjo.2004.046797/047308

9 Dua HS, King AJ, Joseph A. A new classification of ocular surface burns. *British Journal of Ophthalmology* 2001;**85**:1379–83. doi:10.1136/bjo.85.11.1379

10 Morgan SJ. Chemical burns of the eye: causes and management. *British Journal of Ophthalmology* 1987;**71**:854–7.

11 Blackburn J, Levitan EB, MacLennan PA, *et al.* The Epidemiology of Chemical Eye Injuries. *Curr Eye Res* 2012;**37**:787–93. doi:10.3109/02713683.2012.681747

12 Grundlingh J, Payne J, Hassan T. Attacks with corrosive substances are increasing in UK. *BMJ* 2017;**358**:j3640.

13 Ahmed F, Maroof H, Ahmed N, *et al.* Acid attacks_ A new public health pandemic in the west? *International Journal of Surgery* 2017;**48**:32–3. doi:10.1016/j.ijsu.2017.09.073

14 HM Government. *Offensive Weapons Act 2019*. Norwich: : The Stationery Office 2019. http://www.legislation.gov.uk/ukpga/2019/17/pdfs/ukpga_20190017_en.pdf

15 Sentencing Council. **Sentencing guidelines and offences involving corrosive substances**. 2017.https://www.sentencingcouncil.org.uk/news/item/sentencing-guidelines-and-offences-involving-corrosive-substances/ (accessed 3 Sep2018).

16 Davis A, Baldwin A, Hingorani M, *et al.* A review of 145 234 ophthalmic patient episodes lost to follow-up. *Eye (Lond)* 2017;**31**:422–9. doi:10.1038/eye.2016.225

17 King A, David D, Jones HS, *et al.* Factors affecting non-attendance in an ophthalmic outpatient department. *JRSM* 1995;**88**:88–90.

For peer review only

18 Campbell K, Millard A, McCartney G, *et al. Who is least likely to attend? An analysis of outpatient appointment “did not attend”(DNA) data in Scotland*. Edinburgh: : NHS Health Scotland 2015.

19 Blais BR. Treating chemical eye injuries. *Occup Health Saf* 1996;**65**:23–6.

20 Daly M, Tuft SJ, Munro PMG. Acute corneal calcification following chemical injury. *Cornea* 2005;**24**:761–5.

21 Schrage NF, Schlossmacher B, Aschenbernner W, *et al.* Phosphate buffer in alkali eye burns as an inducer of experimental corneal calcification. *Burns* 2001;**27**:459–64.

22 Brodovsky SC, McCarty CA, Snibson G, *et al.* Management of alkali burns : an 11-year retrospective review. *Ophthalmology* 2000;**107**:1829–35.

COMPETING INTERESTS STATEMENT

There is no support from any organisation for the submitted work; no financial relationships with any organisations that might have an interest in the submitted work in the previous three years; and no other relationships or activities that could appear to have influenced the submitted work.

AUTHOR STATEMENT

Searched the literature: JJH, EJC, AJS
Drafted initial protocol: EJC
Gained ethical approval: EJC
Contributed to protocol development and revision: JJH, EJC, AJS
Database design: EJC
Data collection: JJH, EJC
Statistical analysis: JJH
Database management: JJH
Drafted this manuscript: JJH
Critically revised this manuscript: JJH, EJC, AJS
Conceptualization: EJC, AJS
Supervision: AJS

JJH and EJC contributed equally to the preparation of this manuscript.

For peer review only

TRANSPARENCY DECLARATION

The lead author (the manuscript's guarantor) affirms that the manuscript is an honest, accurate, and transparent account of the study being reported; that no important aspects of the study have been omitted; and that any discrepancies from the study as planned (and, if relevant, registered) have been explained.

DATA STATEMENT

There are no additional unpublished data from this study. The datasets generated during and/or analysed during the current study will be available upon request from Dr Jeremy Hoffman (jeremy.hoffman@lshtm.ac.uk). The full data set will be available with all patient identifiable details removed. Datasets will only be available to bona fide scientific investigators. Requests should be made to Dr Jeremy Hoffman in writing detailing the scientific investigators background and intended use for the data. Consideration will be given to all proposed analyses.

LEGENDS FOR FIGURES

Figure 1: Demographics and mode of injury of cases of SOCI (n = 25) presenting to Moorfields Eye Hospital between 1st September 2011 and 31st August 2014.

Figure 2: Initial acute management given in the Emergency Department for patients presenting with SOCI (n = 25) during the three-year study period. PF = preservative free

For peer review only

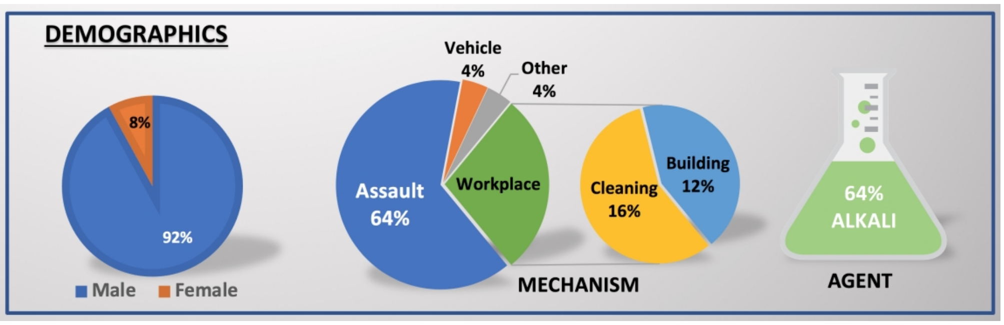

Demographics and mode of injury of cases of SOCI (n = 25) presenting to Moorfields Eye Hospital between 1st September 2011 and 31st August 2014.

235x74mm (300 x 300 DPI)

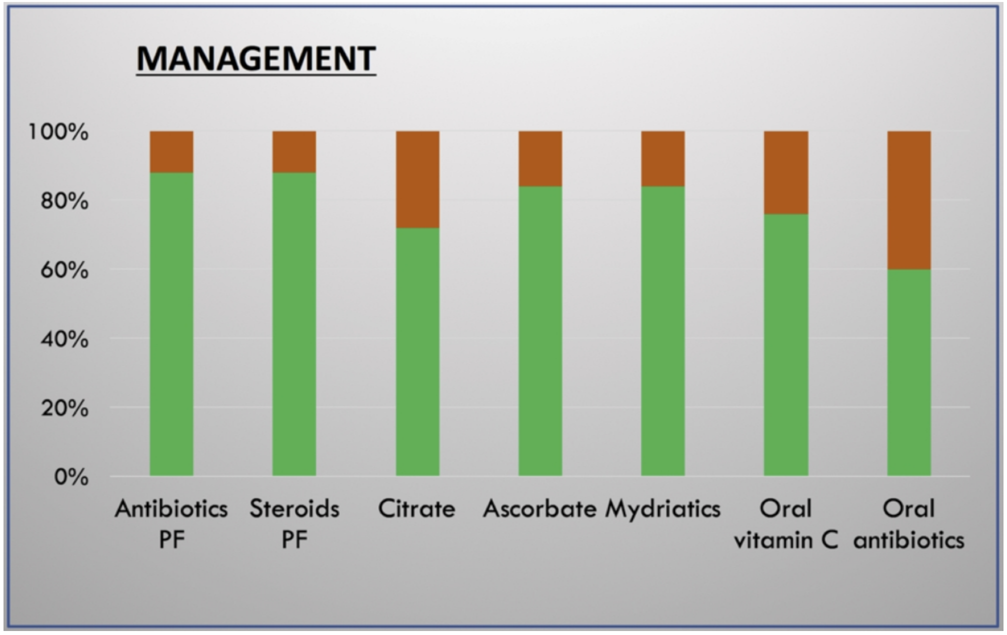

Initial acute management given in the Emergency Department for patients presenting with SOCI (n = 25) during the three-year study period. PF = preservative free

318x200mm (300 x 300 DPI)

Assault-related severe ocular chemical injury at a London ophthalmic referral hospital: a three-year retrospective observational study

Journal:	*BMJ Open*
Manuscript ID	bmjopen-2020-038109.R1
Article Type:	Original research
Date Submitted by the Author:	30-Jul-2020
Complete List of Authors:	Hoffman, Jeremy; Moorfields Eye Hospital NHS Foundation Trust, Accident and Emergency Department; University College London Institute of Ophthalmology,<br>Casswell, Edward; Moorfields Eye Hospital NHS Foundation Trust, Accident and Emergency Department; University College London Institute of Ophthalmology,<br>Shortt, Alex; Moorfields Eye Hospital NHS Foundation Trust; University College London Institute of Ophthalmology,
**Primary Subject Heading**:	Ophthalmology
Secondary Subject Heading:	Public health, Emergency medicine
Keywords:	ACCIDENT & EMERGENCY MEDICINE, OPHTHALMOLOGY, Corneal and external diseases < OPHTHALMOLOGY, PUBLIC HEALTH

SCHOLARONE™
Manuscripts

I, the Submitting Author has the right to grant and does grant on behalf of all authors of the Work (as defined in the below author licence), an exclusive licence and/or a non-exclusive licence for contributions from authors who are: i) UK Crown employees; ii) where BMJ has agreed a CC-BY licence shall apply, and/or iii) in accordance with the terms applicable for US Federal Government officers or employees acting as part of their official duties; on a worldwide, perpetual, irrevocable, royalty-free basis to BMJ Publishing Group Ltd ("BMJ") its licensees and where the relevant Journal is co-owned by BMJ to the co-owners of the Journal, to publish the Work in this journal and any other BMJ products and to exploit all rights, as set out in our licence.

The Submitting Author accepts and understands that any supply made under these terms is made by BMJ to the Submitting Author unless you are acting as an employee on behalf of your employer or a postgraduate student of an affiliated institution which is paying any applicable article publishing charge ("APC") for Open Access articles. Where the Submitting Author wishes to make the Work available on an Open Access basis (and intends to pay the relevant APC), the terms of reuse of such Open Access shall be governed by a Creative Commons licence – details of these licences and which Creative Commons licence will apply to this Work are set out in our licence referred to above.

Other than as permitted in any relevant BMJ Author's Self Archiving Policies, I confirm this Work has not been accepted for publication elsewhere, is not being considered for publication elsewhere and does not duplicate material already published. I confirm all authors consent to publication of this Work and authorise the granting of this licence.

For peer review only

Assault-related severe ocular chemical injury at a London ophthalmic referral hospital: a three-year retrospective observational study

Authors: Jeremy John Hoffman (1, 2)*, Edward Joshua Casswell (1,2)*, Alex John Shortt (1, 2)

*- these authors contributed equally to the preparation of this manuscript

Affiliations:

(1) Moorfields Eye Hospital, London, UK

(2) NIHR Biomedical Research Centre, UCL Institute of Ophthalmology, London, UK

Conflict of interest:

All authors declare there is no conflict of interests.

Corresponding author:

Dr Jeremy Hoffman FRCOphth

UCL Institute of Ophthalmology

11-43 Bath Street

London, UK

EC1V 9EL

jeremy.hoffman@lshtm.ac.uk

Keywords:

Chemical injury, acid attack, corneal burns, ophthalmology, cornea

Word Count:

3728

For peer review only

ABSTRACT

Objectives:

To understand the incidence, causes, management and outcomes of intentional (assault) and unintentional severe ocular chemical injuries (SOCI) at an urban tertiary referral centre in the UK.

Design:

Retrospective observational study.

Setting:

A London tertiary referral ophthalmic centre, Moorfields Eye Hospital.

Participants:

All cases of SOCI presenting between 1st September 2011 and 31st August 2014 were identified. The definition of SOCI was grade 3 or 4 on the Hughes-Roper-Hall classification system. We identified 25 cases (6 in 2011-12, 8 in 2012-13, 11 in 2013-14). Median age was 31.1 years. 23 cases (92%) were male.

Primary and secondary outcome measures:

The primary outcome was the proportion of cases of SOCI caused by assault, per year. Secondary outcome measures included the number of cases of SOCI, injury characteristics and mechanism, initial and long-term management, visual outcome and the need for surgical intervention.

Results:

Between 2011-12, 3/6 cases were due to assault (50%); between 2012-13, 7/8 were due to assault (87.5%); and between 2013-14, 6/11 were due to assault (54.4%). Assault was responsible for 16/25 (64%) cases overall, whilst 8/25 (32%) cases were work-related. The causative agent was known to be alkali in 16/25 (64%), whilst 10/25 (40%) did not complete follow-up. The mean number of clock hours of limbal ischaemia was 5.24 (SD 2.97). 17/25 (68%) were Hughes-Roper-Hall grade 3. Surgical intervention occurred in 1/25. The final BCVA was worse than 6/12 in 11/25 (44%) and was counting fingers in 4/25 (16%).

Conclusions:

Previous studies found that SOCI had a low incidence and that work-related injuries were the most common cause. Our study demonstrates an increasing incidence of SOCI which may be accounted for by a rise in assault using corrosive substances. A high number of patients did not attend regularly for follow-up and visual outcomes from these injuries are poor.

For peer review only

ARTICLE SUMMARY

Strengths and limitations of this study

- This is acase series, spanning three years, from a tertiary ophthalmic hospital in London
- The primary outcome was the proportion of cases of SOCI caused by assault, per year
- The methodology differs to the previous national multicentre prospective (BOSU) study conducted between 2005-06 meaning comparisons should be made with caution

For peer review only

INTRODUCTION

Ocular chemical injury is an ophthalmic emergency and accounts for 11.5-22.1% of ophthalmic trauma.[1] Rapid emergency first-aid management with copious irrigation is crucial, followed by appropriate acute ophthalmic care.[2] Fortunately, most injuries are minor and unintentional.[3] However, severe ocular chemical injuries (SOCI), although infrequent, carry a high risk of developing devastating, long-term sequelae and sight loss if not managed appropriately and promptly.[2]

There are several factors that influence the severity of the injury, including the agent itself (acid or alkali), its strength, length of contact with the eye, volume and concentration. SOCI usually results from strong acid or alkali agents.[4] Alkali injuries are often most severe, as the basic charged molecules can rapidly cross the corneal epithelium and stroma and enter the anterior chamber.[2]

Several grading systems for ocular chemical injuries exist. Hughes noted the relationship between initial presenting clinical signs and prognosis; this was subsequently amended by Ballen and then Roper-Hall.[5-7] Although more recent systems have been developed, the Hughes-Roper-Hall (H-R-H) classification system is easy to use and remember, maintaining its popularity particularly in the ophthalmic emergency department.[8] The H-R-H system divides the injury into four grades. Grade 1 cases are mild with corneal epithelial damage only and no limbal ischaemia with a clear cornea. Grade 2 demonstrates a hazy cornea but with iris details still visible and less than one-third (three clock hours) limbal ischaemia. Grade 3 involves between one-third and one-half limbal ischaemia (three to six clock hours), hazy cornea obscuring iris details and complete epithelial loss. Grade 4, the most severe, presents with an opaque cornea and greater than one-half limbal ischaemia. H-R-H Grades 3 and 4 are considered SOCI.[3] The most recent evolution of burns classification is the Dua classification which includes an assessment of the degree of conjunctival burn involvement.[9]

A surveillance study of SOCI conducted in the UK from December 2005 to November 2006 only identified 12 cases this year, with a minimum estimated incidence of 0.02 per 100,000.[3] They found that 8/12 (66.7%) patients with SOCI were working-age men and half (6/12) occurred at work. Assault accounted for 4/12 (33.3%) of injuries.[3] These findings are supported by previous studies investigating mild, as well as severe ocular chemical injuries.[1,4,10] In one study from the US, similar demographics were reported, although assault only accounted for 2 out of 640 cases (0.31%).[11] A more recent study from the US investigating the epidemiological trends of ocular chemical injuries (including mild cases)

For peer review only

reports a median age of 32, with 56.6% of cases being male. Age-specific incidence rates per 100,000 population were highest amongst individuals aged between 20 and 29, with a mean of 17.4/100,000. This study also reported a high incidence amongst infants and children. The authors did not report whether injuries were intentional or unintentional.[12]

SOCI can either be caused by purposeful human action, defined as an “intentional injury”; chemical assault is an example of this. Other injuries are those that are accidental, referred to as unintentional injuries. In the UK, particularly in metropolitan centres such as London, there has been a rapid rise in chemical assault or so-called “acid” attacks.[13] This increase has received significant attention in the press with a number of high-profile attacks, with the incidence increasing by 65% in 2017 alone.[14] In London, the Metropolitan Police recorded an increase from 162 cases in 2012 to 454 in 2016; this number is higher than all the chemical assaults recorded in India that year.[13] However, a retrospective study from a leading national burns centre in London questions whether this increase that is being reported in the national press is translating into a similar increase in chemical burn presentations.[15]

The aim of this study was to investigate the number and causes of SOCI presentations per year over a three-year period at Moorfields Eye Hospital, a central London tertiary referral eye hospital with a 24-hour ophthalmic emergency department. In addition, this study aimed to describe the demographics, responsible agent, clinical presentation and management of SOCI cases.

For peer review only

MATERIALS AND METHODS

This study was a retrospective, observational study of patients attending Moorfields Eye Hospital Emergency Department with severe ocular chemical injuries. The study received approval from the Moorfields Eye Hospital Research on Anonymised Data (ROAD) Ethics Committee in February 2015 (Ref: ROAD 15/010) and the Moorfields Eye Hospital Clinical Audit Department (Ref: CA15/CED/11). The study was compliant with the Declaration of Helsinki. Formal patient consent was deemed not necessary by the Moorfields ROAD Ethics Committee.

Severe ocular chemical injuries were defined as either grade 3 or 4 on the Hughes-Roper-Hall (H-R-H) classification system.[5,7] The H-R-H classification was chosen over the newer Dua classification to follow the same inclusion criteria as reported in the BOSU Study.[3] Furthermore, this classification is what is currently used and documented in our Emergency Department, with the treatment protocol dependent on the H-R-H grade.

All electronic records of emergency department attendances between 1st September 2011 and 31st August 2014 were searched for the following coded diagnoses “Chemical Injury or Trauma” or “Chemical Trauma” who were coded as either being admitted or followed-up in clinic. Patient records were excluded from review if they were discharged directly from the department without follow-up as these cases would have represented minor injuries. All detailed clinical records of these attendances were then reviewed by one of two ophthalmologists (EC and JH) at Moorfields Eye Hospital. Cases meeting the SOCI definition above were included in the study. Patients with multiple attendances for the same initial event were only included once (i.e. duplicate records were excluded). Patients without H-R-H 3 or 4 were excluded from the study; all other records were included. For any missing data or outlying values, the records were reviewed again by the other ophthalmologist to ensure quality control. Data entry and management was conducted within Microsoft Excel 2016 (Microsoft Corp., Ridgemont, VA), whilst statistical analysis was performed using STATA 15 (StataCorp, College Station, TX). To calculate p-values to determine significance when comparing intentional to non-intentional injuries, means were compared using the t-test, whilst categorical variables were compared using chi-squared calculations.

The primary outcome was the proportion of cases of SOCI caused by assault, per year. Secondary outcomes included the number of cases of SOCI, characteristics and mechanism of the injury, initial and long-term management, visual outcome and the need for surgical intervention. **Patient and Public Involvement**

For peer review only

Following the publication of our research in an open-access, peer reviewed journal, we plan on raising the awareness of the risks of chemical injury to the eye and measures to prevent this from happening, through public awareness campaigns. We would also seek to involve first-responders in disseminating the advice on the emergency treatment of ocular chemical injuries.

RESULTS

There were 242 attendances coded as either "Chemical Injury or Trauma" or "Chemical Trauma" over the study period. Complete clinical records were available and reviewed for 219 individuals, whilst the electronic records were available for 19 cases. There were three duplicate records and one record was unavailable or missing. The retrieval rate for the study was therefore 99.6%. Of the 238 notes reviewed, 25 cases met the inclusion criteria of SOCI.

Demographics and mode of injury

The median age at presentation was 31.1 years, (range 17 – 74, inter-quartile range 27 – 42). Table 1 describes the patient demographics and mode of injury, where 23/25 cases (92%) were male. Intentional injury caused by assault was responsible for 16/25 (64%) cases across the three years, with the remaining 9/25 (36%) classified as unintentional or accidental. Of these, 7/9 (77.8%) of the cases were work-related. Intentional injuries occurred predominantly in public places (including the street, public transport and bars/pubs), accounting for 10/16 (62.5%) injuries. Intentional injuries at home and at work occurred in 2/16 (12.5%) and 1/16 (6.25%) of cases, respectively. Injury with an alkali substance was reported in 16/25 (64%) and unknown in 6/25 (24%).

There were 6 cases of SOCI identified between 2011-12, of which 3/6 were due to assault (50%); 8 between 2012-13, 7/8 due to assault (87.5%); and 11 between 2013-14, 6/11 due to assault (54.4%), Figure 1. During the same period, there were 77,652 encounters in the emergency department in 2011-12; 81,899 in 2012-13; and 85,799 in 2013-14. This gives a presentation rate of SOCI at Moorfields of 0.773 cases per 10,000 encounters in 2011-12; 0.977 cases per 10,000 encounters in 2012-13; and 1.28 cases per 10,000 encounters in 2013-14. There was a mean year-on-year increase of 0.254 cases per 10,000 encounters. There was an approximate increase of 65.6% SOCI encounters from 2011-12 to 2013-14.

For peer review only

Table 1: Patient demographics, mechanism, place of injury and chemical agent responsible for the injury

	n	(%)
**Patients**	25	(100)
Male	23	(92)
Female	2	(8)
**Mechanism and place of injury**		
Intentional - Assault	16	(100)
Public - Street	8	(50)
Public – Public Transport	1	(6.25)
Public – Bar / Pub	1	(6.25)
Work	1	(6.25)
Home	2	(12.5)
Unknown	3	(18.75)
Unintentional	9	(100)
Work - Cleaning	3	(33.3)
Work - Building	3	(33.3)
Work - Vehicle	1	(11.1)
Home - Cleaning	1	(11.1)
Home - Other	1	(11.1)
**Chemical agent**	25	(100)
Acid	1	(4)
Alkali	16	(64)
Other	2	(8)
Unknown	6	(24)

For peer review only

Clinical Presentation

Tables 2 and 3 describes the baseline and follow-up visual acuity, H-R-H grade, surgical management and clinical outcome for our cohort of patients. At presentation, the visual acuity was 6/18 (20/60) or worse in 15/25 (60%) of patients, with 3/25 (12%) of patients 6/60 (20/200) or worse. The mean number of clock hours of limbal ischaemia was 5.24 (SD 2.97). There were 17/25 (68%) cases of H-R-H Grade 3 and 8/25 (32%) cases of H-R-H Grade 4. Time to irrigation data were available in 9/25 (36%) cases: irrigation was performed within one hour of the index event in only 5/9 (55%) of these cases, whilst the mean time to irrigation was 2.6 hours (range 0.5 – 15 hours; SD 4.70).

Management

The initial management instigated in our Emergency Department is described in Figure 2. All patients received a topical antibiotic and steroid at presentation, 22/25 (88%) of whom received preservative-free formulations. Topical citrate and ascorbate were prescribed in 18/25 (72%) and 21/25 (84%) of cases respectively. Oral ascorbate and oral doxycycline were administered to 19/25 (76%) and 15/25 (60%) of patients respectively. The full recommended protocol of preservative free antibiotics and steroids; topical citrate and ascorbate; and oral ascorbate and doxycycline was followed in 5/25 (20%).

Long term management and outcome

Of the 25 patients with H-R-H grade 3 to 4 injuries, 10/25 (40%) failed to comply with standard follow up regimes in our department and were lost to follow-up at various stages of treatment. 2/10 patients did not attend for review beyond one week and were discharged, 6/10 did not attend reviews beyond one month and 2/10 failed to attend appointments between 3 and 12 months, despite being offered alternative appointments, as per hospital policy. The final BCVA (defined here as the later of either the last clinic appointment in the patient records prior to discharge, or one year following the index event) was less than 6/12 (20/40) in 11/25 (44%) of cases. There were 4/25 (16%) of patient with vision at counting fingers or worse at final follow-up, Tables 2 and 3.

Long term complications included keratoconjunctivitis sicca (6/25), persistent epithelial defects (4/25), microbial keratitis (1/25), central pannus (2/25), central corneal scarring (8/25), entropion (1/25) and cataract (1/25), Table 2. Of note, there were no recorded cases of corneal melt, perforation, secondary glaucoma or patients requiring eye removal.

For peer review only

Surgical intervention was performed in 1/25 (4%), who underwent an amniotic membrane graft, a limbal stem cell graft and finally a penetrating keratoplasty. This same patient subsequently developed cataract and underwent uncomplicated phacoemulsification and intra-ocular lens insertion.

Comparison between intentional and unintentional injuries

Table 3 compares the demographics, management and outcomes for patients whose injuries were classified as either intentional (i.e. assault) or unintentional. Intentional injuries occurred solely amongst men: this was found to be statistically significant when compared to unintentional injuries ($p = 0.049$). Unintentional injuries occurred solely at home or at work, whilst the majority of intentional injuries occurred in public places (10/16, 62.5%). These differences in location between the intentional and unintentional injury groups was found to be statistically significant ($p = < 0.001$). Other than these, there was no statistically significant difference identified between the intentional and unintentional injury groups in terms of presenting features, management or outcome.

For peer review only

Table 2: Best corrected visual acuity at presentation and at final follow-up, Hughes-Roper-Hall grade, surgical intervention and corneal sequelae for all patients, and whether patients were lost to follow-up.

Patient	BCVA at presentation	BCVA at final follow-up	H-R-H Grade	Surgical intervention	Long-term complications	Lost to follow-up?
1	6/9	6/4	3	-		No
2	6/18	6/18	3	-	KS, CCS	No
3	6/24	6/6	3	-		No
4	6/6	6/9	3	-	CCS	No
5	6/12	6/5	3	-		DNA at 1/52
6	6/6	6/9	3	-	KS	No
7	6/24	Unknown	3	-		DNA at 1/52
8	6/9	6/12	4	-		DNA at 1/12
9	6/9	6/5	3	-		No
10	6/24	6/6	4	-	KS, CCS, CP	No
11	6/9	6/18	4	-		DNA at 1/12
12	6/18	6/6	3	-	CCS	No
13	6/12	6/12	3	-		DNA at 1/12
14	6/36	6/9	4	-		DNA at 1/12
15	6/24	6/12	4	-		DNA at 1/12
16	6/18	6/9	3	-	KS, CCS	No
17	CF	HM	4	-	PED,	No
18	6/18	6/12	3	-		No
19	4/60	3/60	3	-		DNA at 3/12
20	6/12	HM	3	-	KS, PED, CCS	DNA at 12/12
21	6/36	HM	4	-	KS, PED, MK, CCS, CP, Ent	No
22	6/36	6/6	3	-		DNA at 1/12
23	6/12	6/5	3	-		No
24	3/60	6/12	4	-		No
25	6/18	6/9	3	AMG, LSC, PK, Phaco	PED, CCS, Cat	No

H-R-H, Hughes-Roper-Hall; DNA, Did Not Attend follow-up; AMG, amniotic membrane graft; LSC, limbal stem cell graft; PK, penetrating keratoplasty; Phaco, phacoemulsification and intra-ocular lens insertion; KS, keratoconjunctivitis sicca; CCS, central corneal scar; CP, central pannus; PED, persistent epithelial defect; MK, microbial keratitis; Ent, entropion

For peer review only

Table 3: Comparison of select patient demographics, injury mechanism, place of injury, clinical features, management and outcome between patients sustaining an intentional injury (i.e. assault) and those sustaining an unintentional (accidental) injury

	Intentional (assault) n = 16		Unintentional n = 9		p-value
	n	(%)	n	(%)	
**Patients**	16	(100)	9	(100)	
Male	16	(100)	7	(77.8)	0.049*
Female	0	(0)	2	(22.2)	0.049*
Median age (years)	29.7		38.3		0.0897
**Mechanism and place of injury**					
Injury occurred at home or work	3	(18.75)	9	(100)	<0.001*
Injury occurred in public	10	(62.5)	0	(0)	<0.001*
Alkali injury	10	(62.5)	6	(66.7)	0.835
Acid injury	0	(0)	1	(6.25)	0.174
**Presenting clinical features**					
Presenting BCVA < = 6/18	10	(62.5)	5	(55.6)	0.734
Presenting BCVA < = 6/60	3	(18.8)	0	(0)	0.166
Mean limbal ischaemia	5.75 (SD 2.26)		4.33 (SD 3.94)		0.8693
H-R-H Grade 3	10	(62.5)	7	(77.8)	0.432
H-R-H Grade 4	6	(37.5)	2	(22.2)	0.432
**Management**					
Mean time to irrigation (hours)	5.67 (SD 8.09)		1.17 (SD 0.98)		0.9040
Preservative free antibiotics and steroids	12	(75)	7	(77.8)	0.876
Full treatment protocol	2	(12.5)	3	(33.3)	0.211
**Outcome**					
Patient failed to attend follow-up	6	(37.5)	4	(44.4)	0.734
Surgical intervention	1	(6.25)	0	(0)	0.444
Vision < = 6/12	7	(43.75)	4	(44.4)	0.772
Vision <= CF	3	(18.75)	1	(11.1)	0.699

BCVA, Best Corrected Visual Acuity; H-R-H, Hughes-Roper-Hall Grade; SD, Standard Deviation; * = statistically significant p-value. Statistical testing performed: t-test for means, chi-squared for categorical variables.

For peer review only

DISCUSSION

The number of cases of SOCI presenting to Moorfields Eye Hospital increased year-on-year between 2011 and 2014, at a mean increase of 0.254 cases per 10,000 encounters per year. There was an increase of 65.6% from 2011/12 to 2013/14. The number of cases attributable to assault doubled from 3/6 (50%) to 7/8 (87.5%) between 2011/12 and 2012/13. In 2013/14 there were 6/11 cases of SOCI caused by assault. Overall across the three years, assault was responsible for 16/25 (64%) presentations.

These results contrast to those of the BOSU study from 2005-06.[3] The calculated incidence in 2005 was 0.02 per 100 000, based on the 12 patients reported over the course of the year and assuming a UK population of 60 million. We report that in 2013/14 there were 11 cases of SOCI seen within one tertiary eye unit in Central London. Assuming that Moorfields Eye Hospital serves half of the London population for ophthalmic services and a population of 8.79 million, we estimate that the incidence of SOCI is 0.25 per 100 000 in London. This is more than 10 times greater than the 2005-06 study. Furthermore, we have found that the proportion of injuries due to assault compared to work-related is 64% to 28% respectively; this contrasts to the BOSU results of 33% and 50% respectively. Demographic characteristics between the two studies are similar with SOCI being more common in young males, although we found a greater proportion of males (92% compared to 75%).

The authors of the BOSU study anticipated a maximum of 200-300 cases of SOCI annually in the UK, based on two previous studies.[3,4,10] If our results were extrapolated across the UK with a population of 65.6 million, we would expect approximately 164 case of SOCI annually. This is closer to Macdonald *et al's* anticipated level of cases. However, it must be emphasized that the methodology differs significantly between the two studies: one is a retrospective review of cases at a single tertiary centre whilst the other is a prospective survey conducted across the UK. This means that direct comparisons and any conclusions should be made with caution. It is also likely that the BOSU study may have under-reported the results by up to 25%.[3] With our study being conducted in a tertiary centre, our results may over-estimate the incidence. Furthermore, it is likely that chemical injuries due to assault are higher in cities, and possibly in London, compared to the rest of the UK. We chose to conduct this present study given anecdotal evidence of an increase in SOCI at our centre. As opposed to the prospective methodology used in BOSU surveillance studies, this enables a "snapshot" to gauge trends. We would therefore advocate further nationwide multicentre prospective studies to evaluate the true incidence of SOCI.

For peer review only

A recent study by Bizrah and co-workers from our same institution reported 10 patients with SOCI identified over three months in 2016, of which seven (70%) were attributable to assault.[16] This suggests that the incidence of SOCI continues to increase and that assault-related chemical injuries continue to be a problem, with a similar proportion of assault-related injuries as in our cohort of patients.

The reasons behind the rise in "acid-attacks" are likely multifactorial. In the past, acid attacks were previously related to robberies. Now, with tougher legislation against carrying knives, corrosive substances were seen as a replacement weapon that was harder to detect, with no legislation previously in the UK against carrying it. However, the Offensive Weapons Act 2019, closes this loophole and places restricitons on the sale of corrosive substances and makes it an offensive to have a corrosive substance in a public place. [17] Furthermore, a person found guilty is liable for a fine and/or imprisonment for up to 12 months. This is in addition to sentencing guidelines that classify corrosive substances as "highly dangerous weapons", meaning longer custodial sentences for those found guilty of using a corrosive substance to threaten or assault others.[18] Many of these attacks are related to gangs, with men being twice as likely as women to be the victims.[13] It will be interesting to see if these legislative changes will translate to a reduced incidence of assault-related SOCI and further studies similar to ours and that of Bizrah and co-workers are advocated.[16]

An interesting result from our study is that 40% (10/25) of our patients were lost to follow-up as they did not attend (DNA) subsequent review appointments. This raises several observations. Firstly, this may affect the results of our study. For example, the final visual acuity may be better than we reported if patients with better vision were more likely to DNA. Secondly, although this DNA rate is the same as that reported from a recent study relating to SOCI, [16] this rate is considerably higher than that reported by previous studies of general ophthalmic outpatient attendances in the UK where the reported rate is 12.5-12.9%.[19,20] This is likely to be due to demographics; DNA rates are typically higher in young men, those from deprived backgrounds and those living in urban areas.[21] This patient demographic is likely over-represented in our study population, compared to the general ophthalmic population. When comparing intentional to unintentional injuries, there was no statistically significant difference between the DNA rates between the groups (Table 3), indicating that assault itself was not a predictor for patients who eventually DNA. This finding has important implications for outpatient management: patients attending with SOCI should receive additional counselling on the importance of their follow-up appointments and may benefit from additional reminders via telephone calls, emails or text messages.

For peer review only

The most important step to treating chemical injury and improving the outcome is immediate irrigation – “dilution is the solution”.[1,4,22] This is ideally done immediately at the scene following the exposure, continued by the paramedical team in the ambulance on the way to hospital and repeated again at presentation until the pH has normalised. This should be performed with normal saline where available, but if this is not available, clean water should be used. In our study, this was performed in 5/9 (55%) of patients within one hour of the injury, less than the 75% reported by MacDonald *et al,* although it is important to consider that we only had data for 36% participants.[3] There is therefore a need to improve people’s awareness of the importance of irrigation. Given the shift in the mode of injury from workplace-related injury, for which there already exists robust health and safety legislation and awareness, to assault-related, we suggest raising awareness amongst people at risk of falling victim to “acid-attacks” through appropriately targeted advertising campaigns.

The aim of early medical management is to reduce inflammation and promote corneal re-epithelialisation, and prevent acute complications such as infection or corneal melt.[2] Treatment should avoid the use of preserved medications, particularly those containing phosphates, as this can precipitate acute corneal calcification.[23,24] Acute treatment therefore involves preservative-free topical steroids and antibiotics to reduce inflammation and prevent infection. Topical citrate and ascorbate helps to prevent corneal melts.[25] Oral doxycycline can reduce the inflammation and the risk of melt.[2] Oral ascorbate 1g daily can also prevent collagen lysis, promote healing and prevent corneal melts.[2] The protocol at Moorfields Eye Hospital is that all patients with SOCI receive preservative-free steroid and antibiotic, topical ascorbate and citrate, oral ascorbate and doxycycline and cycloplegia. In our study, whilst all patients received topical steroid and antibiotics, 76% (19/25) received preservative-free formulation. This has improved since the BOSU study where only half (6/12) of patients received preservative-free formulations.[3] Topical citrate and oral doxycycline were prescribed in 48% of patients (12/25), compared to 25% (3/12) of patients in the BOSU study.[3]

Surgical intervention was low in our study, with only one patient (1/25, 4%) undergoing surgical treatment. This same patient underwent multiple forms of ocular surface reconstructive surgery. This low surgical rate of intervention seen in our study compares to 50% (6/12) of cases in the BOSU study, of which half of these had more than one surgical procedure.[3] Reasons for this are unclear, but it is possible that the high DNA rate may have resulted in a falsely low rate of surgical intervention, with patients being lost to follow up before surgical intervention was appropriate.

For peer review only

Final vision was reduced to worse than 6/12 in 44% of cases, which is marginally better compared to 50% reported by MacDonald et al.[3] The rate of very severe visual loss (worse than 6/60) was 16% in our study, comparable to 16.7% in the BOSU study.[3] This suggests that on the whole, visual prognosis from SOCI remains poor, with the potential for significant morbidity and ocular sequelae as a result of the injury.

There are a number of limitations to our study. Firstly, as mentioned above, the methodology between our study and that of the BOSU study are different, meaning direct comparisons should be interpreted with a degree of caution. Secondly, although we report a large case series relative to other other studies, the absolute number of patients in our study, despite it running for three years, is only 25, as SOCI is a rare condition. This may make statistical analysis challenging, as our sample size may be insufficiently powered to detect significance. Thirdly, with this study being a retrospective review of case notes, the accuracy of the data collected was dependent on the record-keeping of the attending clinician. This was minimised by cross-checking the paper clinical records with online records that include patient letters and surgical interventions, to ensure we did not miss any outcome data. We are therefore confident that we were able to gather all the relevant clinical data for this cohort.

This study suggests that there has been a recent increase in the incidence of severe ocular chemical injury in a central London tertiary eye hospital, with assault now responsible for between one half and just under two-thirds of cases. It is likely that SOCI is more common than has previously been reported, particularly in metropolitan areas. This increase may be a result of the recent increase in “acid attacks”, although further work is necessary to establish causality. Regardless, there is a need to reverse the increase in “acid” attacks through stronger legislation to act as a deterrent. There was a lower than expected level of initial irrigation and so public awareness of how to manage chemical injury could be improved. Similarly, first responders and emergency department personnel should be reminded of the importance of copious irrigation and onward referral to a specialist ophthalmic unit. Ophthalmologists attending these cases should follow an evidenced-based protocol and be aware of the risk of patients not attending their follow-up appointments and plan accordingly. An updated nationwide surveillance study is warranted to enable direct comparisons to what was seen nearly 15 years ago.

Funding:

Jeremy Hoffman was supported by the National Institute for Health Research (NIHR) Biomedical Research Centre based at Moorfields Eye Hospital NHS Foundation Trust and UCL Institute of Ophthalmology (ACF-2015-18-020). Alex Shortt was supported by a

For peer review only

Wellcome Trust Intermediate Clinical Fellowship based at the UCL Institute of Ophthalmology and the UCL Institute of Immunity and Transplantation (102804/Z/13/Z). Ed Casswell was supported through a grant from the Friends of Moorfields Eye Hospital Charity. The views expressed are those of the author(s) and not necessarily those of the Wellcome Trust, NHS, the NIHR or the Department of Health. There was no specific funding for this research.

For peer review only

References

1 Wagoner MD. Chemical injuries of the eye: current concepts in pathophysiology and therapy. *Survey of Ophthalmology* 1997;**41**:275–313.

2 Tuft SJ, Shortt AJ. Surgical rehabilitation following severe ocular burns. *Eye (Lond)* 2009;**23**:1966–71. doi:10.1038/eye.2008.414

3 Macdonald ECA, Cauchi PA, Azuara-Blanco A, *et al.* Surveillance of severe chemical corneal injuries in the UK. *British Journal of Ophthalmology* 2009;**93**:1177–80. doi:10.1136/bjo.2008.154831

4 Kuckelkorn R, Schrage N, Keller G, *et al.* Emergency treatment of chemical and thermal eye burns. *Acta Ophthalmol Scand* 2002;**80**:4–10.

5 Hughes WF. Alkali burns of the eye; clinical and pathologic course. *Arch Ophthal* 1946;**36**:189–214.

6 Ballen PH. Treatment of chemical burns to the eye. *Eye Ear Nose Throat Mon* 1964;**43**:57–61.

7 Roper-Hall MJ. Thermal and chemical burns. *Trans Ophthalmol Soc U K* 1965;**85**:631–53.

8 Harun S, Srinivasan S, Hollingworth K, *et al.* Modification of classification of ocular chemical injuries. *British Journal of Ophthalmology* 2004;**88**:1353–4–authorreply1354–5. doi:10.1136/bjo.2004.046797/047308

9 Dua HS, King AJ, Joseph A. A new classification of ocular surface burns. *British Journal of Ophthalmology* 2001;**85**:1379–83. doi:10.1136/bjo.85.11.1379

10 Morgan SJ. Chemical burns of the eye: causes and management. *British Journal of Ophthalmology* 1987;**71**:854–7.

11 Blackburn J, Levitan EB, MacLennan PA, *et al.* The Epidemiology of Chemical Eye Injuries. *Curr Eye Res* 2012;**37**:787–93. doi:10.3109/02713683.2012.681747

12 Haring RS, Sheffield ID, Channa R, *et al.* Epidemiologic Trends of Chemical Ocular Burns in the United States. *JAMA Ophthalmol* 2016;**134**:1119–24. doi:10.1001/jamaophthalmol.2016.2645

13 Grundlingh J, Payne J, Hassan T. Attacks with corrosive substances are increasing in UK. *BMJ* 2017;**358**:j3640.

14 Ahmed F, Maroof H, Ahmed N, *et al.* Acid attacks_ A new public health pandemic in the west? *International Journal of Surgery* 2017;**48**:32–3. doi:10.1016/j.ijsu.2017.09.073

15 Miller R, Pywell S, Leon-Villapalos J, *et al.* Chemical burn assaults: Is the media coverage representative? *Burns* 2018;**44**:1021–2. doi:10.1016/j.burns.2018.01.004

16 Bizrah M, Yusuf A, Ahmad S. Adherence to Treatment and Follow-Up in Patients with Severe Chemical Eye Burns. *Ophthalmology and Therapy* 2019;**8**:251–9. doi:10.1007/s40123-019-0173-y

For peer review only

17 HM Government. *Offensive Weapons Act 2019*. Norwich: : The Stationery Office 2019. http://www.legislation.gov.uk/ukpga/2019/17/pdfs/ukpga_20190017_en.pdf

18 Sentencing Council. **Sentencing guidelines and offences involving corrosive substances**. 2017.https://www.sentencingcouncil.org.uk/news/item/sentencing-guidelines-and-offences-involving-corrosive-substances/ (accessed 3 Sep2018).

19 Davis A, Baldwin A, Hingorani M, *et al.* A review of 145 234 ophthalmic patient episodes lost to follow-up. *Eye (Lond)* 2017;**31**:422–9. doi:10.1038/eye.2016.225

20 King A, David D, Jones HS, *et al.* Factors affecting non-attendance in an ophthalmic outpatient department. *JRSM* 1995;**88**:88–90.

21 Campbell K, Millard A, McCartney G, *et al. Who is least likely to attend? An analysis of outpatient appointment "did not attend"(DNA) data in Scotland*. Edinburgh: : NHS Health Scotland 2015.

22 Blais BR. Treating chemical eye injuries. *Occup Health Saf* 1996;**65**:23–6.

23 Daly M, Tuft SJ, Munro PMG. Acute corneal calcification following chemical injury. *Cornea* 2005;**24**:761–5.

24 Schrage NF, Schlossmacher B, Aschenbernner W, *et al.* Phosphate buffer in alkali eye burns as an inducer of experimental corneal calcification. *Burns* 2001;**27**:459–64.

25 Brodovsky SC, McCarty CA, Snibson G, *et al.* Management of alkali burns : an 11-year retrospective review. *Ophthalmology* 2000;**107**:1829–35.

COMPETING INTERESTS STATEMENT

There is no support from any organisation for the submitted work; no financial relationships with any organisations that might have an interest in the submitted work in the previous three years; and no other relationships or activities that could appear to have influenced the submitted work.

AUTHOR STATEMENT

Searched the literature: JJH, EJC, AJS

Drafted initial protocol: EJC

Gained ethical approval: EJC

Contributed to protocol development and revision: JJH, EJC, AJS

Database design: EJC

Data collection: JJH, EJC

Statistical analysis: JJH

Database management: JJH

For peer review only

Drafted this manuscript: JJH

Critically revised this manuscript: JJH, EJC, AJS

Conceptualization: EJC, AJS

Supervision: AJS

JJH and EJC contributed equally to the preparation of this manuscript.

TRANSPARENCY DECLARATION

The lead author (the manuscript's guarantor) affirms that the manuscript is an honest, accurate, and transparent account of the study being reported; that no important aspects of the study have been omitted; and that any discrepancies from the study as planned (and, if relevant, registered) have been explained.

DATA STATEMENT

There are no additional unpublished data from this study. The datasets generated during and/or analysed during the current study will be available upon request from Dr Jeremy Hoffman (jeremy.hoffman@lshtm.ac.uk). The full data set will be available with all patient identifiable details removed. Datasets will only be available to bona fide scientific investigators. Requests should be made to Dr Jeremy Hoffman in writing detailing the scientific investigators background and intended use for the data. Consideration will be given to all proposed analyses.

LEGENDS FOR FIGURES

Figure 1: Patients with assault-related severe ocular chemical injury presenting to Moorfields Eye Hospital between 2011 and 2014 as a proportion of severe ocular chemical injury presentations.

Figure 2: Initial acute management given in the Emergency Department for patients presenting with severe ocular chemical injury (n = 25) during the three-year study period. PF = preservative free

For peer review only

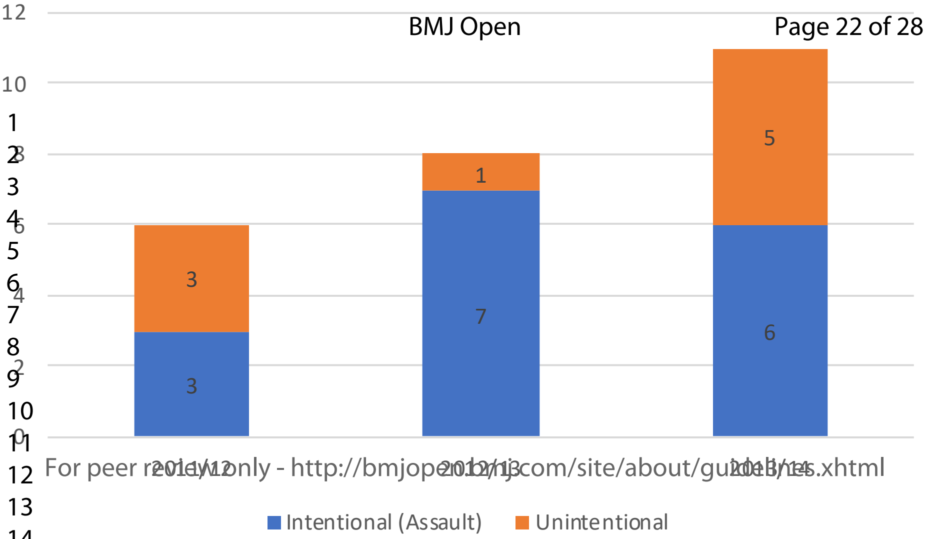
12
10
8
6
4
2
0
3
3
7
1
6
5
2011/12
2012/13
2013/14
Intentional (Assault)
Unintentional

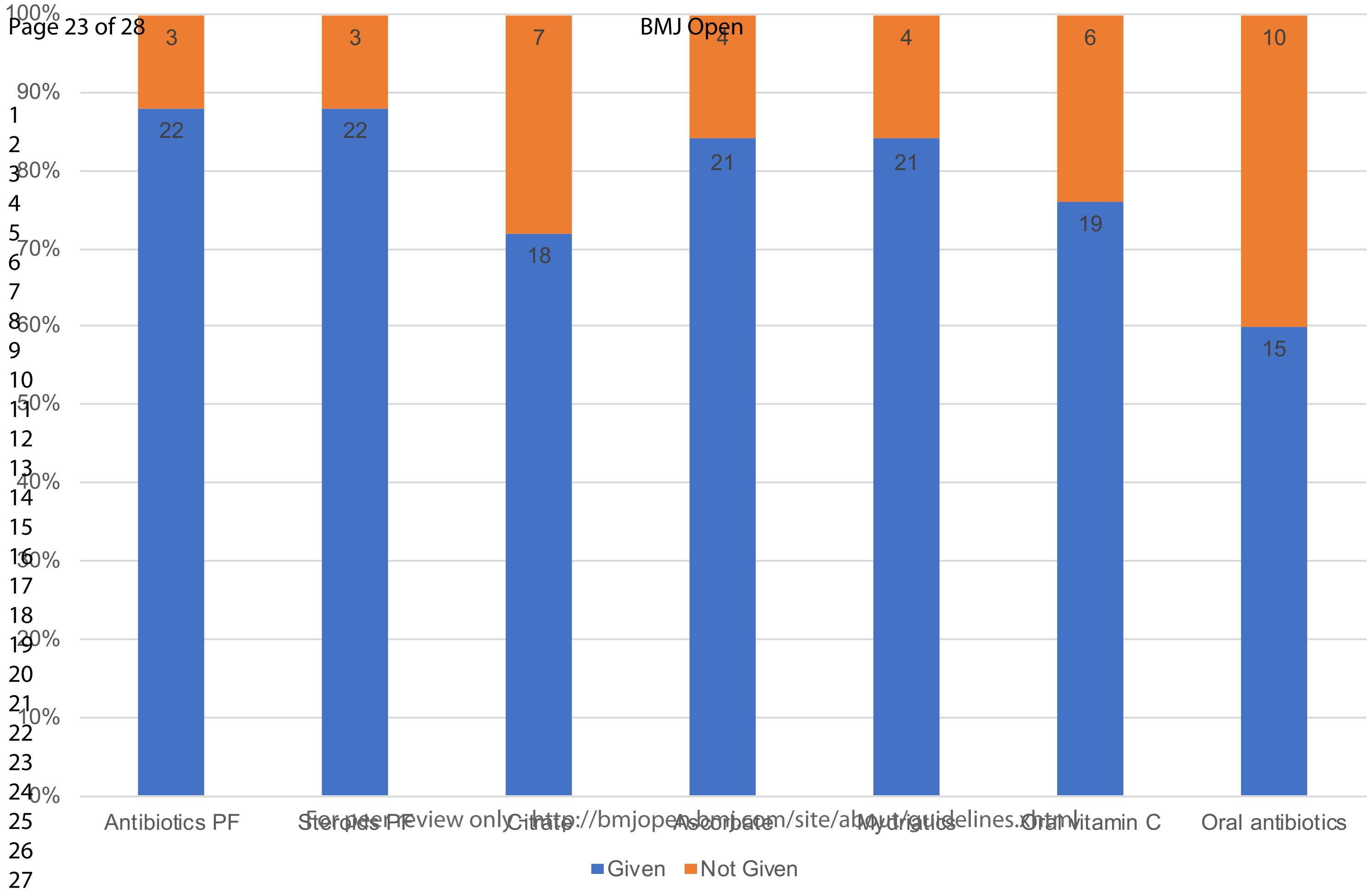

For peer review only - http://bmjopen.bmj.com/site/about/guidelines.xhtml

The RECORD statement – checklist of items, extended from the STROBE statement, that should be reported in observational studies using routinely collected health data.

	Item No.	STROBE items	Location in manuscript where items are reported	RECORD items	Location in manuscript where items are reported
**Title and abstract**					
	1	(a) Indicate the study's design with a commonly used term in the title or the abstract (b) Provide in the abstract an informative and balanced summary of what was done and what was found	Title and Abstract (Pages 1-2)	RECORD 1.1: The type of data used should be specified in the title or abstract. When possible, the name of the databases used should be included.<br><br>RECORD 1.2: If applicable, the geographic region and timeframe within which the study took place should be reported in the title or abstract.<br><br>RECORD 1.3: If linkage between databases was conducted for the study, this should be clearly stated in the title or abstract.	Title and Abstract (Pages 1-2)
**Introduction**					
Background rationale	2	Explain the scientific background and rationale for the investigation being reported	Introduction section, pages 4-5		
Objectives	3	State specific objectives, including any prespecified hypotheses	Page 5, final paragraph		
**Methods**					
Study Design	4	Present key elements of study design early in the paper	Methods section, pages 6-7		
Setting	5	Describe the setting, locations, and relevant dates, including periods of recruitment, exposure, follow-up, and data collection	Methods section, page 6, first paragraph		

For peer review only

Participants	6	*(a) Cohort study* - Give the eligibility criteria, and the sources and methods of selection of participants. Describe methods of follow-up<br>*Case-control study* - Give the eligibility criteria, and the sources and methods of case ascertainment and control selection. Give the rationale for the choice of cases and controls<br>*Cross-sectional study* - Give the eligibility criteria, and the sources and methods of selection of participants<br><br>*(b) Cohort study* - For matched studies, give matching criteria and number of exposed and unexposed<br>*Case-control study* - For matched studies, give matching criteria and the number of controls per case	Methods section, page 6, paragraphs 2 and 3	RECORD 6.1: The methods of study population selection (such as codes or algorithms used to identify subjects) should be listed in detail. If this is not possible, an explanation should be provided.<br><br>RECORD 6.2: Any validation studies of the codes or algorithms used to select the population should be referenced. If validation was conducted for this study and not published elsewhere, detailed methods and results should be provided.<br><br>RECORD 6.3: If the study involved linkage of databases, consider use of a flow diagram or other graphical display to demonstrate the data linkage process, including the number of individuals with linked data at each stage.	Methods section, page 6, paragraphs 2 and 3<br><br>N/A<br><br>N/A
Variables	7	Clearly define all outcomes, exposures, predictors, potential confounders, and effect modifiers. Give diagnostic criteria, if applicable.	Methods section, page 6, paragraph 4	RECORD 7.1: A complete list of codes and algorithms used to classify exposures, outcomes, confounders, and effect modifiers should be provided. If these cannot be reported, an explanation should be provided.	N/A
Data sources/ measurement	8	For each variable of interest, give sources of data and details of methods of assessment (measurement).<br>Describe comparability of assessment methods if there is more than one group	Methods section, page 6, paragraph 3		

For peer review only

Bias	9	Describe any efforts to address potential sources of bias	N/A		
Study size	10	Explain how the study size was arrived at	N/A		
Quantitative variables	11	Explain how quantitative variables were handled in the analyses. If applicable, describe which groupings were chosen, and why	N/A		
Statistical methods	12	(a) Describe all statistical methods, including those used to control for confounding<br>(b) Describe any methods used to examine subgroups and interactions<br>(c) Explain how missing data were addressed<br>(d) *Cohort study* - If applicable, explain how loss to follow-up was addressed<br>*Case-control study* - If applicable, explain how matching of cases and controls was addressed<br>*Cross-sectional study* - If applicable, describe analytical methods taking account of sampling strategy<br>(e) Describe any sensitivity analyses	Methods section, paragraph 3		
Data access and cleaning methods		..		RECORD 12.1: Authors should describe the extent to which the investigators had access to the database population used to create the study population.	Methods section, paragraph 3

For peer review only

				RECORD 12.2: Authors should provide information on the data cleaning methods used in the study.	
Linkage		..		RECORD 12.3: State whether the study included person-level, institutional-level, or other data linkage across two or more databases. The methods of linkage and methods of linkage quality evaluation should be provided.	N/A
**Results**					
Participants	13	(a) Report the numbers of individuals at each stage of the study (*e.g.*, numbers potentially eligible, examined for eligibility, confirmed eligible, included in the study, completing follow-up, and analysed)<br>(b) Give reasons for non-participation at each stage.<br>(c) Consider use of a flow diagram	Results section, page 7, first paragraph	RECORD 13.1: Describe in detail the selection of the persons included in the study (*i.e.,* study population selection) including filtering based on data quality, data availability and linkage. The selection of included persons can be described in the text and/or by means of the study flow diagram.	Methods and results section, pages 6-7
Descriptive data	14	(a) Give characteristics of study participants (*e.g.*, demographic, clinical, social) and information on exposures and potential confounders<br>(b) Indicate the number of participants with missing data for each variable of interest<br>(c) *Cohort study* - summarise follow-up time (*e.g.*, average and total amount)	Results section, page 7, first paragraph<br><br>Results section, page 7, second paragraph		
Outcome data	15	*Cohort study* - Report numbers of outcome events or summary measures over time<br>*Case-control study* - Report numbers in each exposure	Results section, page 7, second paragraph		

For peer review only

		category, or summary measures of exposure<br>*Cross-sectional study* - Report numbers of outcome events or summary measures			
Main results	16	(a) Give unadjusted estimates and, if applicable, confounder-adjusted estimates and their precision (e.g., 95% confidence interval). Make clear which confounders were adjusted for and why they were included<br>(b) Report category boundaries when continuous variables were categorized<br>(c) If relevant, consider translating estimates of relative risk into absolute risk for a meaningful time period	Results section, page 7, first paragraph in results section.<br>Results section, third paragraph.<br><br>Tables 1-3<br><br>Figures 1-2<br><br>Results section, pages 9 - 10		
Other analyses	17	Report other analyses done—e.g., analyses of subgroups and interactions, and sensitivity analyses	N/A		
**Discussion**					
Key results	18	Summarise key results with reference to study objectives	Page 13, paragraph 1		
Limitations	19	Discuss limitations of the study, taking into account sources of potential bias or imprecision. Discuss both direction and magnitude of any potential bias	Page 13, paragraph 3 and page 16, paragraph 2	RECORD 19.1: Discuss the implications of using data that were not created or collected to answer the specific research question(s). Include discussion of misclassification bias, unmeasured confounding, missing data, and changing eligibility over time, as they pertain to the study being reported.	N/A
Interpretation	20	Give a cautious overall interpretation of results considering objectives,	Discussion section, pages 13-16		

For peer review only

		limitations, multiplicity of analyses, results from similar studies, and other relevant evidence			
Generalisability	21	Discuss the generalisability (external validity) of the study results	Page 16, final paragraph of discussion		
**Other Information**					
Funding	22	Give the source of funding and the role of the funders for the present study and, if applicable, for the original study on which the present article is based	Page 16		
Accessibility of protocol, raw data, and programming code		..		RECORD 22.1: Authors should provide information on how to access any supplemental information such as the study protocol, raw data, or programming code.	Data sharing statement provided page 20

*Reference: Benchimol EI, Smeeth L, Guttmann A, Harron K, Moher D, Petersen I, Sørensen HT, von Elm E, Langan SM, the RECORD Working Committee. The REporting of studies Conducted using Observational Routinely-collected health Data (RECORD) Statement. *PLoS Medicine* 2015; in press.

*Checklist is protected under Creative Commons Attribution (CC BY) license.

For peer review only

BMJ Open

Assault-related severe ocular chemical injury at a London ophthalmic referral hospital: a three-year retrospective observational study

Journal:	*BMJ Open*
Manuscript ID	bmjopen-2020-038109.R2
Article Type:	Original research
Date Submitted by the Author:	26-Aug-2020
Complete List of Authors:	Hoffman, Jeremy; Moorfields Eye Hospital NHS Foundation Trust, Accident and Emergency Department; University College London Institute of Ophthalmology,<br>Casswell, Edward; Moorfields Eye Hospital NHS Foundation Trust, Accident and Emergency Department; University College London Institute of Ophthalmology,<br>Shortt, Alex; Moorfields Eye Hospital NHS Foundation Trust; University College London Institute of Ophthalmology,
**Primary Subject Heading**:	Ophthalmology
Secondary Subject Heading:	Public health, Emergency medicine
Keywords:	ACCIDENT & EMERGENCY MEDICINE, OPHTHALMOLOGY, Corneal and external diseases < OPHTHALMOLOGY, PUBLIC HEALTH

SCHOLARONE™
Manuscripts

I, the Submitting Author has the right to grant and does grant on behalf of all authors of the Work (as defined in the below author licence), an exclusive licence and/or a non-exclusive licence for contributions from authors who are: i) UK Crown employees; ii) where BMJ has agreed a CC-BY licence shall apply, and/or iii) in accordance with the terms applicable for US Federal Government officers or employees acting as part of their official duties; on a worldwide, perpetual, irrevocable, royalty-free basis to BMJ Publishing Group Ltd ("BMJ") its licensees and where the relevant Journal is co-owned by BMJ to the co-owners of the Journal, to publish the Work in this journal and any other BMJ products and to exploit all rights, as set out in our licence.

The Submitting Author accepts and understands that any supply made under these terms is made by BMJ to the Submitting Author unless you are acting as an employee on behalf of your employer or a postgraduate student of an affiliated institution which is paying any applicable article publishing charge ("APC") for Open Access articles. Where the Submitting Author wishes to make the Work available on an Open Access basis (and intends to pay the relevant APC), the terms of reuse of such Open Access shall be governed by a Creative Commons licence – details of these licences and which Creative Commons licence will apply to this Work are set out in our licence referred to above.

Other than as permitted in any relevant BMJ Author's Self Archiving Policies, I confirm this Work has not been accepted for publication elsewhere, is not being considered for publication elsewhere and does not duplicate material already published. I confirm all authors consent to publication of this Work and authorise the granting of this licence.

For peer review only

Assault-related severe ocular chemical injury at a London ophthalmic referral hospital: a three-year retrospective observational study

Authors: Jeremy John Hoffman (1, 2)*, Edward Joshua Casswell (1,2)*, Alex John Shortt (1, 2)

*- these authors contributed equally to the preparation of this manuscript

Affiliations:

(1) Moorfields Eye Hospital, London, UK

(2) NIHR Biomedical Research Centre, UCL Institute of Ophthalmology, London, UK

Conflict of interest:

All authors declare there is no conflict of interests.

Corresponding author:

Dr Jeremy Hoffman FRCOphth

UCL Institute of Ophthalmology

11-43 Bath Street

London, UK

EC1V 9EL

jeremy.hoffman@lshtm.ac.uk

Keywords:

Chemical injury, acid attack, corneal burns, ophthalmology, cornea

Word Count:

3993

For peer review only

ABSTRACT

Objectives:

To understand the incidence, causes, management and outcomes of intentional (assault) and unintentional severe ocular chemical injuries (SOCI) at an urban tertiary referral centre in the UK.

Design:

Retrospective observational study.

Setting:

A London tertiary referral ophthalmic centre, Moorfields Eye Hospital.

Participants:

All cases of SOCI presenting between 1st September 2011 and 31st August 2014 were identified. The definition of SOCI was grade 3 or 4 on the Hughes-Roper-Hall classification system. We identified 25 cases (6 in 2011-12, 8 in 2012-13, 11 in 2013-14). Median age was 31.1 years. 23 cases (92%) were male.

Primary and secondary outcome measures:

The primary outcome was the proportion of cases of SOCI caused by assault, per year. Secondary outcome measures included the number of cases of SOCI, injury characteristics and mechanism, initial and long-term management, visual outcome and the need for surgical intervention.

Results:

Between 2011-12, 3/6 cases were due to assault (50%); between 2012-13, 7/8 were due to assault (87.5%); and between 2013-14, 6/11 were due to assault (54.4%). Assault was responsible for 16/25 (64%) cases overall, whilst 8/25 (32%) cases were work-related. The causative agent was known to be alkali in 16/25 (64%), whilst 10/25 (40%) did not complete follow-up. The mean number of clock hours of limbal ischaemia was 5.24 (SD 2.97). 17/25 (68%) were Hughes-Roper-Hall grade 3. Surgical intervention occurred in 1/25. The final BCVA was worse than 6/12 in 11/25 (44%) and was counting fingers in 4/25 (16%).

Conclusions:

Previous studies found that SOCI had a low incidence and that work-related injuries were the most common cause. Our study demonstrates an increasing incidence of SOCI which may be accounted for by a rise in assault using corrosive substances. A high number of patients did not attend regularly for follow-up and visual outcomes from these injuries are poor.

For peer review only

ARTICLE SUMMARY

Strengths and limitations of this study

- This is acase series, spanning three years, from a tertiary ophthalmic hospital in London
- The primary outcome was the proportion of cases of SOCI caused by assault, per year
- The methodology differs to the previous national multicentre prospective (BOSU) study conducted between 2005-06 meaning comparisons should be made with caution

For peer review only

INTRODUCTION

Ocular chemical injury is an ophthalmic emergency and accounts for 11.5-22.1% of ophthalmic trauma.[1] Rapid emergency first-aid management with copious irrigation is crucial, followed by appropriate acute ophthalmic care.[2] Fortunately, most injuries are minor and unintentional.[3] However, severe ocular chemical injuries (SOCI), although infrequent, carry a high risk of developing devastating, long-term sequelae and sight loss if not managed appropriately and promptly.[2]

There are several factors that influence the severity of the injury, including the agent itself (acid or alkali), its strength, length of contact with the eye, volume and concentration. SOCI usually results from strong acid or alkali agents.[4] Alkali injuries are often most severe, as the basic charged molecules can rapidly cross the corneal epithelium and stroma and enter the anterior chamber.[2]

Several grading systems for ocular chemical injuries exist. Hughes noted the relationship between initial presenting clinical signs and prognosis; this was subsequently amended by Ballen and then Roper-Hall.[5-7] Although more recent systems have been developed, the Hughes-Roper-Hall (H-R-H) classification system is easy to use and remember, maintaining its popularity particularly in the ophthalmic emergency department.[8] The H-R-H system divides the injury into four grades. Grade 1 cases are mild with corneal epithelial damage only and no limbal ischaemia with a clear cornea. Grade 2 demonstrates a hazy cornea but with iris details still visible and less than one-third (three clock hours) limbal ischaemia. Grade 3 involves between one-third and one-half limbal ischaemia (three to six clock hours), hazy cornea obscuring iris details and complete epithelial loss. Grade 4, the most severe, presents with an opaque cornea and greater than one-half limbal ischaemia. H-R-H Grades 3 and 4 are considered SOCI.[3] The most recent evolution of burns classification is the Dua classification which includes an assessment of the degree of conjunctival burn involvement.[9]

A surveillance study of SOCI conducted in the UK from December 2005 to November 2006 only identified 12 cases this year, with a minimum estimated incidence of 0.02 per 100,000.[3] They found that 8/12 (66.7%) patients with SOCI were working-age men and half (6/12) occurred at work. Assault accounted for 4/12 (33.3%) of injuries.[3] These findings are supported by previous studies investigating mild, as well as severe ocular chemical injuries.[1,4,10] In one study from the US, similar demographics were reported, although assault only accounted for 2 out of 640 cases (0.31%).[11] A more recent study from the US investigating the epidemiological trends of ocular chemical injuries (including mild cases)

For peer review only

reports a median age of 32, with 56.6% of cases being male. Age-specific incidence rates per 100,000 population were highest amongst individuals aged between 20 and 29, with a mean of 17.4/100,000. This study also reported a high incidence amongst infants and children. The authors did not report whether injuries were intentional or unintentional.[12]

SOCI can either be caused by purposeful human action, defined as an “intentional injury”; chemical assault is an example of this. Other injuries are those that are accidental, referred to as unintentional injuries. In the UK, particularly in metropolitan centres such as London, there has been a rapid rise in chemical assault or so-called “acid” attacks.[13] This increase has received significant attention in the press with a number of high-profile attacks, with the incidence increasing by 65% in 2017 alone.[14] In London, the Metropolitan Police recorded an increase from 162 cases in 2012 to 454 in 2016; this number is higher than all the chemical assaults recorded in India that year.[13] However, a retrospective study from a leading national burns centre in London questions whether this increase that is being reported in the national press is translating into a similar increase in chemical burn presentations.[15]

The aim of this study was to investigate the number and causes of SOCI presentations per year over a three-year period at Moorfields Eye Hospital, a central London tertiary referral eye hospital with a 24-hour ophthalmic emergency department. In addition, this study aimed to describe the demographics, responsible agent, clinical presentation and management of SOCI cases.

For peer review only

MATERIALS AND METHODS

This study was a retrospective, observational study of patients attending Moorfields Eye Hospital Emergency Department with severe ocular chemical injuries. The study received approval from the Moorfields Eye Hospital Research on Anonymised Data (ROAD) Ethics Committee in February 2015 (Ref: ROAD 15/010) and the Moorfields Eye Hospital Clinical Audit Department (Ref: CA15/CED/11). The study was compliant with the Declaration of Helsinki. Formal patient consent was deemed not necessary by the Moorfields ROAD Ethics Committee.

Severe ocular chemical injuries were defined as either grade 3 or 4 on the Hughes-Roper-Hall (H-R-H) classification system.[5,7] The H-R-H classification was chosen over the newer Dua classification to follow the same inclusion criteria as reported in the BOSU Study.[3] Furthermore, this classification is what is currently used and documented in our Emergency Department, with the treatment protocol dependent on the H-R-H grade.

All electronic records of emergency department attendances between 1st September 2011 and 31st August 2014 were searched for the following coded diagnoses "Chemical Injury or Trauma" or "Chemical Trauma" who were coded as either being admitted or followed-up in clinic. Patient records were excluded from review if they were discharged directly from the department without follow-up as these cases would have represented minor injuries. All detailed clinical records of these attendances were then reviewed by one of two ophthalmologists (EC and JH) at Moorfields Eye Hospital. Cases meeting the SOCI definition above were included in the study. Patients with multiple attendances for the same initial event were only included once (i.e. duplicate records were excluded). Patients without H-R-H 3 or 4 were excluded from the study; all other records were included. For any missing data or outlying values, the records were reviewed again by the other ophthalmologist to ensure quality control. Data entry and management was conducted within Microsoft Excel 2016 (Microsoft Corp., Ridgemont, VA), whilst statistical analysis was performed using STATA 15 (StataCorp, College Station, TX). To calculate p-values to determine significance when comparing intentional to non-intentional injuries, means were compared using the t-test, whilst categorical variables were compared using chi-squared calculations.

The primary outcome was the proportion of cases of SOCI caused by assault, per year. Secondary outcomes included the number of cases of SOCI, characteristics and mechanism of the injury, initial and long-term management, visual outcome and the need for surgical intervention.

For peer review only

Patient and Public Involvement

Following the publication of our research in an open-access, peer reviewed journal, we plan on raising the awareness of the risks of chemical injury to the eye and measures to prevent this from happening, through public awareness campaigns. We would also seek to involve first-responders in disseminating the advice on the emergency treatment of ocular chemical injuries.

RESULTS

There were 242 attendances coded as either “Chemical Injury or Trauma” or “Chemical Trauma” over the study period. Complete clinical records were available and reviewed for 219 individuals, whilst the electronic records were available for 19 cases. There were three duplicate records and one record was unavailable or missing. The retrieval rate for the study was therefore 99.6%. Of the 238 notes reviewed, 25 cases met the inclusion criteria of SOCI.

Demographics and mode of injury

The median age at presentation was 31.1 years, (range 17 – 74, inter-quartile range 27 – 42). Table 1 describes the patient demographics and mode of injury, where 23/25 cases (92%) were male. Intentional injury caused by assault was responsible for 16/25 (64%) cases across the three years, with the remaining 9/25 (36%) classified as unintentional or accidental. Of these, 7/9 (77.8%) of the cases were work-related. Intentional injuries occurred predominantly in public places (including the street, public transport and bars/pubs), accounting for 10/16 (62.5%) injuries. Intentional injuries at home and at work occurred in 2/16 (12.5%) and 1/16 (6.25%) of cases, respectively. Injury with an alkali substance was reported in 16/25 (64%) and unknown in 6/25 (24%).

There were 6 cases of SOCI identified between 2011-12, of which 3/6 were due to assault (50%); 8 between 2012-13, 7/8 due to assault (87.5%); and 11 between 2013-14, 6/11 due to assault (54.4%), Figure 1. During the same period, there were 77,652 encounters in the emergency department in 2011-12; 81,899 in 2012-13; and 85,799 in 2013-14. This gives a presentation rate of SOCI at Moorfields of 0.773 cases per 10,000 encounters in 2011-12; 0.977 cases per 10,000 encounters in 2012-13; and 1.28 cases per 10,000 encounters in 2013-14. There was a mean year-on-year increase of 0.254 cases per 10,000 encounters. There was an approximate increase of 65.6% SOCI encounters from 2011-12 to 2013-14.

For peer review only

Table 1: Patient demographics, mechanism, place of injury and chemical agent responsible for the injury

	n	(%)
**Patients**	25	(100)
Male	23	(92)
Female	2	(8)
**Mechanism and place of injury**		
Intentional - Assault	16	(100)
Public - Street	8	(50)
Public – Public Transport	1	(6.25)
Public – Bar / Pub	1	(6.25)
Work	1	(6.25)
Home	2	(12.5)
Unknown	3	(18.75)
Unintentional	9	(100)
Work - Cleaning	3	(33.3)
Work - Building	3	(33.3)
Work - Vehicle	1	(11.1)
Home - Cleaning	1	(11.1)
Home - Other	1	(11.1)
**Chemical agent**	25	(100)
Acid	1	(4)
Alkali	16	(64)
Other	2	(8)
Unknown	6	(24)

For peer review only

Clinical Presentation

Tables 2 and 3 describes the baseline and follow-up visual acuity, H-R-H grade, surgical management and clinical outcome for our cohort of patients. At presentation, the visual acuity was 6/18 (20/60) or worse in 15/25 (60%) of patients, with 3/25 (12%) of patients 6/60 (20/200) or worse. The mean number of clock hours of limbal ischaemia was 5.24 (SD 2.97). There were 17/25 (68%) cases of H-R-H Grade 3 and 8/25 (32%) cases of H-R-H Grade 4. Time to irrigation data were available in 9/25 (36%) cases: irrigation was performed within one hour of the index event in only 5/9 (55%) of these cases, whilst the mean time to irrigation was 2.6 hours (range 0.5 – 15 hours; SD 4.70).

Management

The initial management instigated in our Emergency Department is described in Figure 2. All patients received a topical antibiotic and steroid at presentation, 22/25 (88%) of whom received preservative-free formulations. Topical citrate and ascorbate were prescribed in 18/25 (72%) and 21/25 (84%) of cases respectively. Oral ascorbate and oral doxycycline were administered to 19/25 (76%) and 15/25 (60%) of patients respectively. The full recommended protocol of preservative free antibiotics and steroids; topical citrate and ascorbate; and oral ascorbate and doxycycline was followed in 5/25 (20%).

Long term management and outcome

Of the 25 patients with H-R-H grade 3 to 4 injuries, 10/25 (40%) failed to comply with standard follow up regimes in our department and were lost to follow-up at various stages of treatment. 2/10 patients did not attend for review beyond one week and were discharged, 6/10 did not attend reviews beyond one month and 2/10 failed to attend appointments between 3 and 12 months, despite being offered alternative appointments, as per hospital policy. The final BCVA (defined here as the later of either the last clinic appointment in the patient records prior to discharge, or one year following the index event) was less than 6/12 (20/40) in 11/25 (44%) of cases. There were 4/25 (16%) of patient with vision at counting fingers or worse at final follow-up, Tables 2 and 3.

Long term complications included keratoconjunctivitis sicca (6/25), persistent epithelial defects (4/25), microbial keratitis (1/25), central pannus (2/25), central corneal scarring (8/25), entropion (1/25) and cataract (1/25), Table 2. Of note, there were no recorded cases of corneal melt, perforation, secondary glaucoma or patients requiring eye removal.

For peer review only

Surgical intervention was performed in 1/25 (4%), who underwent an amniotic membrane graft, a limbal stem cell graft and finally a penetrating keratoplasty. This same patient subsequently developed cataract and underwent uncomplicated phacoemulsification and intra-ocular lens insertion.

Comparison between intentional and unintentional injuries

Table 3 compares the demographics, management and outcomes for patients whose injuries were classified as either intentional (i.e. assault) or unintentional. Intentional injuries occurred solely amongst men, although this was not found to be statistically significant using Fisher's exact test (p = 0.12), likely due to the small sample size. Unintentional injuries occurred solely at home or at work, whilst the majority of intentional injuries occurred in public places (10/16, 62.5%). These differences in location between the intentional and unintentional injury groups was found to be statistically significant (p = 0.003 and p <0.001 for injury in public and at home or work, respectively). Other than these, there was no statistically significant difference identified between the intentional and unintentional injury groups in terms of presenting features, management or outcome.

For peer review only

Table 2: Best corrected visual acuity at presentation and at final follow-up, Hughes-Roper-Hall grade, surgical intervention and corneal sequelae for all patients, and whether patients were lost to follow-up.

Patient	BCVA at presentation	BCVA at final follow-up	H-R-H Grade	Surgical intervention	Long-term complications	Lost to follow-up?
1	6/9	6/4	3	-		No
2	6/18	6/18	3	-	KS, CCS	No
3	6/24	6/6	3	-		No
4	6/6	6/9	3	-	CCS	No
5	6/12	6/5	3	-		DNA at 1/52
6	6/6	6/9	3	-	KS	No
7	6/24	Unknown	3	-		DNA at 1/52
8	6/9	6/12	4	-		DNA at 1/12
9	6/9	6/5	3	-		No
10	6/24	6/6	4	-	KS, CCS, CP	No
11	6/9	6/18	4	-		DNA at 1/12
12	6/18	6/6	3	-	CCS	No
13	6/12	6/12	3	-		DNA at 1/12
14	6/36	6/9	4	-		DNA at 1/12
15	6/24	6/12	4	-		DNA at 1/12
16	6/18	6/9	3	-	KS, CCS	No
17	CF	HM	4	-	PED,	No
18	6/18	6/12	3	-		No
19	4/60	3/60	3	-		DNA at 3/12
20	6/12	HM	3	-	KS, PED, CCS	DNA at 12/12
21	6/36	HM	4	-	KS, PED, MK, CCS, CP, Ent	No
22	6/36	6/6	3	-		DNA at 1/12
23	6/12	6/5	3	-		No
24	3/60	6/12	4	-		No
25	6/18	6/9	3	AMG, LSC, PK, Phaco	PED, CCS, Cat	No

H-R-H, Hughes-Roper-Hall; DNA, Did Not Attend follow-up; AMG, amniotic membrane graft; LSC, limbal stem cell graft; PK, penetrating keratoplasty; Phaco, phacoemulsification and intra-ocular lens insertion; KS, keratoconjunctivitis sicca; CCS, central corneal scar; CP, central pannus; PED, persistent epithelial defect; MK, microbial keratitis; Ent, entropion

For peer review only

Table 3: Comparison of select patient demographics, injury mechanism, place of injury, clinical features, management and outcome between patients sustaining an intentional injury (i.e. assault) and those sustaining an unintentional (accidental) injury

	**Intentional (assault) n = 16**		**Unintentional n = 9**		**p-value**
	**n**	**(%)**	**n**	**(%)**	
**Patients**	16	(100)	9	(100)	
Male	16	(100)	7	(77.8)	0.12
Female	0	(0)	2	(22.2)	
Median age (years)	29.7		38.3		0.0897
**Mechanism and place of injury**					
Injury occurred at home or work	3	(18.75)	9	(100)	<0.001*
Injury occurred in public	10	(62.5)	0	(0)	0.003*
Alkali injury	10	(62.5)	6	(66.7)	1.00
Acid injury	0	(0)	1	(6.25)	0.360
**Presenting clinical features**					
Presenting BCVA < = 6/12	10	(62.5)	5	(55.6)	1.00
Presenting BCVA < = 6/60	3	(18.8)	0	(0)	0.280
Mean limbal ischaemia	5.75 (SD 2.26)		4.33 (SD 3.94)		0.8293 ‡
H-R-H Grade 3	10	(62.5)	7	(77.8)	0.661
H-R-H Grade 4	6	(37.5)	2	(22.2)	0.661
**Management**					
Mean time to irrigation (hours)	5.67 (SD 8.09)		1.17 (SD 0.98)		0.7819 ‡
Preservative free antibiotics and steroids	12	(75)	7	(77.8)	1.00
Full treatment protocol	2	(12.5)	3	(33.3)	0.312
**Outcome**					
Patient failed to attend follow-up	6	(37.5)	4	(44.4)	1.00
Surgical intervention	1	(6.25)	0	(0)	1.00
Vision < = 6/12	7	(43.75)	4	(44.4)	1.00
Vision <= CF	3	(18.75)	1	(11.1)	1.00

BCVA, Best Corrected Visual Acuity; H-R-H, Hughes-Roper-Hall Grade; SD, Standard Deviation; * = statistically significant p-value. Statistical testing performed: t-test for means if normally distributed; if variances are unequal then adjusted using Welch's formula (‡); Fisher's exact for categorical variables.

For peer review only

DISCUSSION

The number of cases of SOCI presenting to Moorfields Eye Hospital increased year-on-year between 2011 and 2014, at a mean increase of 0.254 cases per 10,000 encounters per year. There was an increase of 65.6% from 2011/12 to 2013/14. The number of cases attributable to assault doubled from 3/6 (50%) to 7/8 (87.5%) between 2011/12 and 2012/13. In 2013/14 there were 6/11 cases of SOCI caused by assault. Overall across the three years, assault was responsible for 16/25 (64%) presentations.

These results contrast to those of the BOSU study from 2005-06.[3] The calculated incidence in 2005 was 0.02 per 100 000, based on the 12 patients reported over the course of the year and assuming a UK population of 60 million. We report that in 2013/14 there were 11 cases of SOCI seen within one tertiary eye unit in Central London. Assuming that Moorfields Eye Hospital serves half of the London population for ophthalmic services and a population of 8.79 million, we estimate that the incidence of SOCI is 0.25 per 100 000 in London. This is more than 10 times greater than the 2005-06 study. Furthermore, we have found that the proportion of injuries due to assault compared to work-related is 64% to 28% respectively; this contrasts to the BOSU results of 33% and 50% respectively. Demographic characteristics between the two studies are similar with SOCI being more common in young males, although we found a greater proportion of males (92% compared to 75%).

The authors of the BOSU study anticipated a maximum of 200-300 cases of SOCI annually in the UK, based on two previous studies.[3,4,10] If our results were extrapolated across the UK with a population of 65.6 million, we would expect approximately 164 case of SOCI annually. This is closer to Macdonald *et al's* anticipated level of cases. However, it must be emphasized that the methodology differs significantly between the two studies: one is a retrospective review of cases at a single tertiary centre whilst the other is a prospective survey conducted across the UK. This means that direct comparisons and any conclusions should be made with caution. It is also likely that the BOSU study may have under-reported the results by up to 25%.[3] With our study being conducted in a tertiary centre, our results may over-estimate the incidence. Furthermore, it is likely that chemical injuries due to assault are higher in cities, and possibly in London, compared to the rest of the UK. We chose to conduct this present study given anecdotal evidence of an increase in SOCI at our centre. As opposed to the prospective methodology used in BOSU surveillance studies, this enables a "snapshot" to gauge trends. We would therefore advocate further nationwide multicentre prospective studies to evaluate the true incidence of SOCI.

For peer review only

A recent study by Bizrah and co-workers from our same institution reported 10 patients with SOCI identified over three months in 2016, of which seven (70%) were attributable to assault.[16] This suggests that the incidence of SOCI continues to increase and that assault-related chemical injuries continue to be a problem, with a similar proportion of assault-related injuries as in our cohort of patients.

The reasons behind the rise in "acid-attacks" are likely multifactorial. In the past, acid attacks were previously related to robberies. Now, with tougher legislation against carrying knives, corrosive substances were seen as a replacement weapon that was harder to detect, with no legislation previously in the UK against carrying it. However, the Offensive Weapons Act 2019, closes this loophole and places restricitons on the sale of corrosive substances and makes it an offensive to have a corrosive substance in a public place. [17] Furthermore, a person found guilty is liable for a fine and/or imprisonment for up to 12 months. This is in addition to sentencing guidelines that classify corrosive substances as "highly dangerous weapons", meaning longer custodial sentences for those found guilty of using a corrosive substance to threaten or assault others.[18] Many of these attacks are related to gangs, with men being twice as likely as women to be the victims.[13] It will be interesting to see if these legislative changes will translate to a reduced incidence of assault-related SOCI and further studies similar to ours and that of Bizrah and co-workers are advocated.[16]

An interesting result from our study is that 40% (10/25) of our patients were lost to follow-up as they did not attend (DNA) subsequent review appointments. This raises several observations. Firstly, this may affect the results of our study. For example, the final visual acuity may be better than we reported if patients with better vision were more likely to DNA. Secondly, although this DNA rate is the same as that reported from a recent study relating to SOCI, [16] this rate is considerably higher than that reported by previous studies of general ophthalmic outpatient attendances in the UK where the reported rate is 12.5-12.9%.[19,20] This is likely to be due to demographics; DNA rates are typically higher in young men, those from deprived backgrounds and those living in urban areas.[21] This patient demographic is likely over-represented in our study population, compared to the general ophthalmic population. When comparing intentional to unintentional injuries, there was no statistically significant difference between the DNA rates between the groups (Table 3), indicating that assault itself was not a predictor for patients who eventually DNA. This finding has important implications for outpatient management: patients attending with SOCI should receive additional counselling on the importance of their follow-up appointments and may benefit from additional reminders via telephone calls, emails or text messages.

For peer review only

The most important step to treating chemical injury and improving the outcome is immediate irrigation – "dilution is the solution".[1,4,22] This is ideally done immediately at the scene following the exposure, continued by the paramedical team in the ambulance on the way to hospital and repeated again at presentation until the pH has normalised. This should be performed with normal saline where available, but if this is not available, clean water should be used. In our study, this was performed in 5/9 (55%) of patients within one hour of the injury, less than the 75% reported by MacDonald *et al,* although it is important to consider that we only had data for 36% participants.[3] There is therefore a need to improve people's awareness of the importance of irrigation. Given the shift in the mode of injury from workplace-related injury, for which there already exists robust health and safety legislation and awareness, to assault-related, we suggest raising awareness amongst people at risk of falling victim to "acid-attacks" through appropriately targeted advertising campaigns.

The aim of early medical management is to reduce inflammation and promote corneal re-epithelialisation, and prevent acute complications such as infection or corneal melt.[2] Treatment should avoid the use of preserved medications, particularly those containing phosphates, as this can precipitate acute corneal calcification.[23,24] Acute treatment therefore involves preservative-free topical steroids and antibiotics to reduce inflammation and prevent infection. Topical citrate and ascorbate helps to prevent corneal melts.[25] Oral doxycycline can reduce the inflammation and the risk of melt.[2] Oral ascorbate 1g daily can also prevent collagen lysis, promote healing and prevent corneal melts.[2] The protocol at Moorfields Eye Hospital is that all patients with SOCI receive preservative-free steroid and antibiotic, topical ascorbate and citrate, oral ascorbate and doxycycline and cycloplegia. In our study, whilst all patients received topical steroid and antibiotics, 76% (19/25) received preservative-free formulation. This has improved since the BOSU study where only half (6/12) of patients received preservative-free formulations.[3] Topical citrate and oral doxycycline were prescribed in 48% of patients (12/25), compared to 25% (3/12) of patients in the BOSU study.[3]

Surgical intervention was low in our study, with only one patient (1/25, 4%) undergoing surgical treatment. This same patient underwent multiple forms of ocular surface reconstructive surgery. This low surgical rate of intervention seen in our study compares to 50% (6/12) of cases in the BOSU study, of which half of these had more than one surgical procedure.[3] Reasons for this are unclear, but it is possible that the high DNA rate may have resulted in a falsely low rate of surgical intervention, with patients being lost to follow up before surgical intervention was appropriate.

For peer review only

Final vision was reduced to worse than 6/12 in 44% of cases, which is marginally better compared to 50% reported by MacDonald et al.[3] The rate of very severe visual loss (worse than 6/60) was 16% in our study, comparable to 16.7% in the BOSU study.[3] This suggests that on the whole, visual prognosis from SOCI remains poor, with the potential for significant morbidity and ocular sequelae as a result of the injury.

There are a number of limitations to our study. Firstly, as mentioned above, the methodology between our study and that of the BOSU study are different, meaning direct comparisons should be interpreted with a degree of caution. Secondly, although we report a large case series relative to other other studies, the absolute number of patients in our study, despite it running for three years, is only 25, as SOCI is a rare condition. This may make statistical analysis challenging, as our sample size may be insufficiently powered to detect significance. Thirdly, with this study being a retrospective review of case notes, the accuracy of the data collected was dependent on the record-keeping of the attending clinician. This was minimised by cross-checking the paper clinical records with online records that include patient letters and surgical interventions, to ensure we did not miss any outcome data. We are therefore confident that we were able to gather all the relevant clinical data for this cohort. Finally, the data from this study is from 2011-2014 and may not necessarily be representative of what is currently being seen, particularly given there have been new legislative changes since this data was collected, introduced in 2019.[17] Until that date, intentional injuries caused by corrosive substances had been increasing, suggesting our results represent the start of this becoming a significant problem in the UK and in particular the London region. This is supported by the results form a London-based study that reported 70% of SOCI were caused by intentional injury in 2016.[16] Data on the number of assaults caused by corrosive substances since the legislative changes have yet to be published; once this is available a subsequent study looking at SOCI at our institution would be helpful to see the current trend.

This study suggests that there has been a recent increase in the incidence of severe ocular chemical injury in a central London tertiary eye hospital, with assault now responsible for between one half and just under two-thirds of cases. It is likely that SOCI is more common than has previously been reported, particularly in metropolitan areas. This increase may be a result of the recent increase in “acid attacks”, although further work is necessary to establish causality. Regardless, there is a need to reverse the increase in “acid” attacks through stronger legislation to act as a deterrent. There was a lower than expected level of initial irrigation and so public awareness of how to manage chemical injury could be improved. Similarly, first responders and emergency department personnel should be reminded of the importance of copious irrigation and onward referral to a specialist ophthalmic unit.

For peer review only

Ophthalmologists attending these cases should follow an evidenced-based protocol and be aware of the risk of patients not attending their follow-up appointments and plan accordingly. An updated nationwide surveillance study is warranted to enable direct comparisons to what was seen nearly 15 years ago.

Funding:

Jeremy Hoffman was supported by the National Institute for Health Research (NIHR) Biomedical Research Centre based at Moorfields Eye Hospital NHS Foundation Trust and UCL Institute of Ophthalmology (ACF-2015-18-020). Alex Shortt was supported by a Wellcome Trust Intermediate Clinical Fellowship based at the UCL Institute of Ophthalmology and the UCL Institute of Immunity and Transplantation (102804/Z/13/Z). Ed Casswell was supported through a grant from the Friends of Moorfields Eye Hospital Charity. The views expressed are those of the author(s) and not necessarily those of the Wellcome Trust, NHS, the NIHR or the Department of Health. There was no specific funding for this research.

For peer review only

References

1 Wagoner MD. Chemical injuries of the eye: current concepts in pathophysiology and therapy. *Survey of Ophthalmology* 1997;**41**:275–313.

2 Tuft SJ, Shortt AJ. Surgical rehabilitation following severe ocular burns. *Eye (Lond)* 2009;**23**:1966–71. doi:10.1038/eye.2008.414

3 Macdonald ECA, Cauchi PA, Azuara-Blanco A, *et al.* Surveillance of severe chemical corneal injuries in the UK. *British Journal of Ophthalmology* 2009;**93**:1177–80. doi:10.1136/bjo.2008.154831

4 Kuckelkorn R, Schrage N, Keller G, *et al.* Emergency treatment of chemical and thermal eye burns. *Acta Ophthalmol Scand* 2002;**80**:4–10.

5 Hughes WF. Alkali burns of the eye; clinical and pathologic course. *Arch Ophthal* 1946;**36**:189–214.

6 Ballen PH. Treatment of chemical burns to the eye. *Eye Ear Nose Throat Mon* 1964;**43**:57–61.

7 Roper-Hall MJ. Thermal and chemical burns. *Trans Ophthalmol Soc U K* 1965;**85**:631–53.

8 Harun S, Srinivasan S, Hollingworth K, *et al.* Modification of classification of ocular chemical injuries. *British Journal of Ophthalmology* 2004;**88**:1353–4–authorreply1354–5. doi:10.1136/bjo.2004.046797/047308

9 Dua HS, King AJ, Joseph A. A new classification of ocular surface burns. *British Journal of Ophthalmology* 2001;**85**:1379–83. doi:10.1136/bjo.85.11.1379

10 Morgan SJ. Chemical burns of the eye: causes and management. *British Journal of Ophthalmology* 1987;**71**:854–7.

11 Blackburn J, Levitan EB, MacLennan PA, *et al.* The Epidemiology of Chemical Eye Injuries. *Curr Eye Res* 2012;**37**:787–93. doi:10.3109/02713683.2012.681747

12 Haring RS, Sheffield ID, Channa R, *et al.* Epidemiologic Trends of Chemical Ocular Burns in the United States. *JAMA Ophthalmol* 2016;**134**:1119–24. doi:10.1001/jamaophthalmol.2016.2645

13 Grundlingh J, Payne J, Hassan T. Attacks with corrosive substances are increasing in UK. *BMJ* 2017;**358**:j3640.

14 Ahmed F, Maroof H, Ahmed N, *et al.* Acid attacks_ A new public health pandemic in the west? *International Journal of Surgery* 2017;**48**:32–3. doi:10.1016/j.ijsu.2017.09.073

15 Miller R, Pywell S, Leon-Villapalos J, *et al.* Chemical burn assaults: Is the media coverage representative? *Burns* 2018;**44**:1021–2. doi:10.1016/j.burns.2018.01.004

16 Bizrah M, Yusuf A, Ahmad S. Adherence to Treatment and Follow-Up in Patients with Severe Chemical Eye Burns. *Ophthalmology and Therapy* 2019;**8**:251–9. doi:10.1007/s40123-019-0173-y

For peer review only

17 HM Government. *Offensive Weapons Act 2019*. Norwich: : The Stationery Office 2019. http://www.legislation.gov.uk/ukpga/2019/17/pdfs/ukpga_20190017_en.pdf

18 Sentencing Council. **Sentencing guidelines and offences involving corrosive substances**. 2017.https://www.sentencingcouncil.org.uk/news/item/sentencing-guidelines-and-offences-involving-corrosive-substances/ (accessed 3 Sep2018).

19 Davis A, Baldwin A, Hingorani M, *et al.* A review of 145 234 ophthalmic patient episodes lost to follow-up. *Eye (Lond)* 2017;**31**:422–9. doi:10.1038/eye.2016.225

20 King A, David D, Jones HS, *et al.* Factors affecting non-attendance in an ophthalmic outpatient department. *JRSM* 1995;**88**:88–90.

21 Campbell K, Millard A, McCartney G, *et al. Who is least likely to attend? An analysis of outpatient appointment "did not attend"(DNA) data in Scotland*. Edinburgh: : NHS Health Scotland 2015.

22 Blais BR. Treating chemical eye injuries. *Occup Health Saf* 1996;**65**:23–6.

23 Daly M, Tuft SJ, Munro PMG. Acute corneal calcification following chemical injury. *Cornea* 2005;**24**:761–5.

24 Schrage NF, Schlossmacher B, Aschenbernner W, *et al.* Phosphate buffer in alkali eye burns as an inducer of experimental corneal calcification. *Burns* 2001;**27**:459–64.

25 Brodovsky SC, McCarty CA, Snibson G, *et al.* Management of alkali burns : an 11-year retrospective review. *Ophthalmology* 2000;**107**:1829–35.

COMPETING INTERESTS STATEMENT

There is no support from any organisation for the submitted work; no financial relationships with any organisations that might have an interest in the submitted work in the previous three years; and no other relationships or activities that could appear to have influenced the submitted work.

AUTHOR STATEMENT

Searched the literature: JJH, EJC, AJS

Drafted initial protocol: EJC

Gained ethical approval: EJC

Contributed to protocol development and revision: JJH, EJC, AJS

Database design: EJC

Data collection: JJH, EJC

Statistical analysis: JJH

Database management: JJH

For peer review only

Drafted this manuscript: JJH

Critically revised this manuscript: JJH, EJC, AJS

Conceptualization: EJC, AJS

Supervision: AJS

JJH and EJC contributed equally to the preparation of this manuscript.

TRANSPARENCY DECLARATION

The lead author (the manuscript's guarantor) affirms that the manuscript is an honest, accurate, and transparent account of the study being reported; that no important aspects of the study have been omitted; and that any discrepancies from the study as planned (and, if relevant, registered) have been explained.

DATA STATEMENT

There are no additional unpublished data from this study. The datasets generated during and/or analysed during the current study will be available upon request from Dr Jeremy Hoffman (jeremy.hoffman@lshtm.ac.uk). The full data set will be available with all patient identifiable details removed. Datasets will only be available to bona fide scientific investigators. Requests should be made to Dr Jeremy Hoffman in writing detailing the scientific investigators background and intended use for the data. Consideration will be given to all proposed analyses.

LEGENDS FOR FIGURES

Figure 1: Patients with assault-related severe ocular chemical injury presenting to Moorfields Eye Hospital between 2011 and 2014 as a proportion of severe ocular chemical injury presentations.

Figure 2: Initial acute management given in the Emergency Department for patients presenting with severe ocular chemical injury (n = 25) during the three-year study period. PF = preservative free

For peer review only

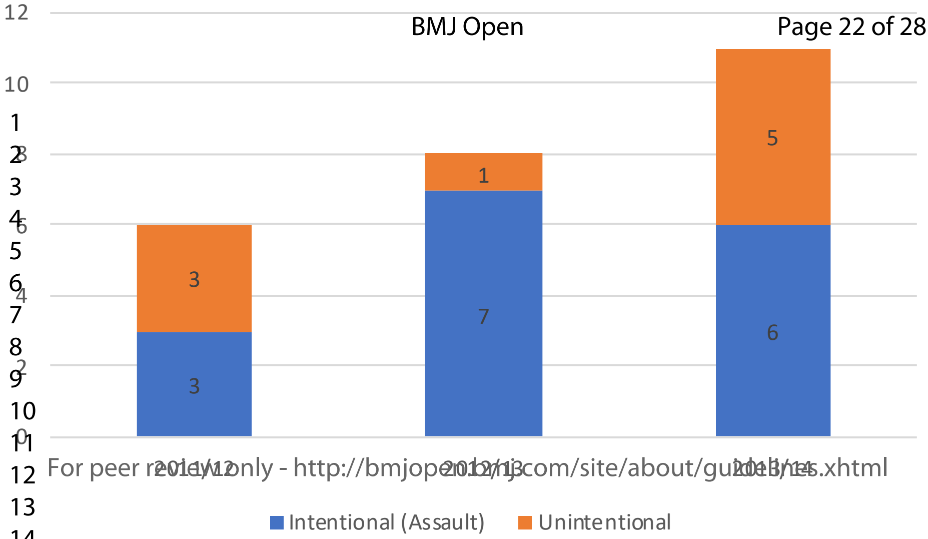
12
10
8
6
4
2
0
3
3
7
1
6
5
2011/12
2012/13
2013/14
Intentional (Assault)
Unintentional

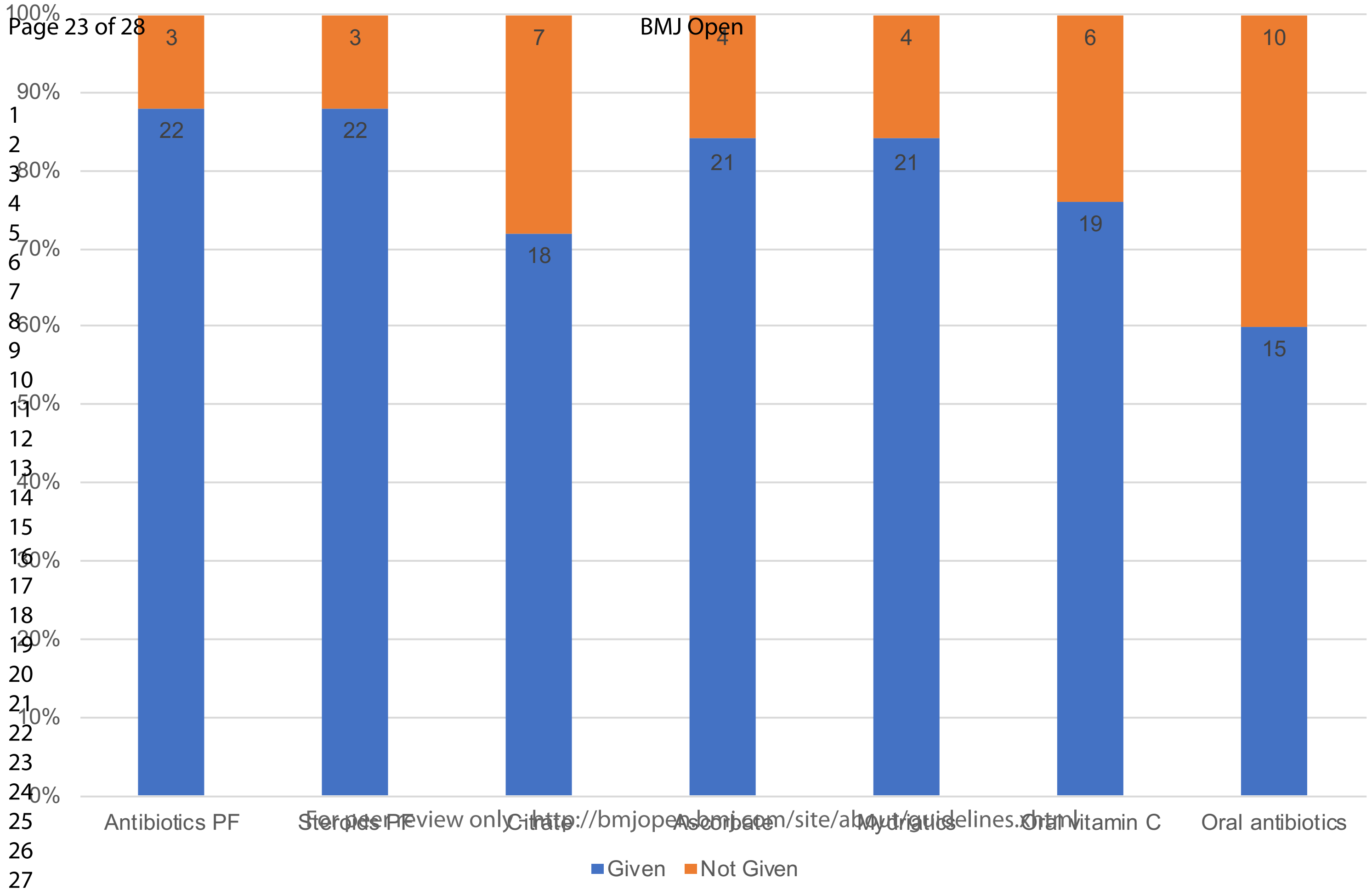

For peer review only - http://bmjopen.bmj.com/site/about/guidelines.xhtml

The RECORD statement – checklist of items, extended from the STROBE statement, that should be reported in observational studies using routinely collected health data.

	Item No.	STROBE items	Location in manuscript where items are reported	RECORD items	Location in manuscript where items are reported
**Title and abstract**					
	1	(a) Indicate the study's design with a commonly used term in the title or the abstract (b) Provide in the abstract an informative and balanced summary of what was done and what was found	Title and Abstract (Pages 1-2)	RECORD 1.1: The type of data used should be specified in the title or abstract. When possible, the name of the databases used should be included.<br><br>RECORD 1.2: If applicable, the geographic region and timeframe within which the study took place should be reported in the title or abstract.<br><br>RECORD 1.3: If linkage between databases was conducted for the study, this should be clearly stated in the title or abstract.	Title and Abstract (Pages 1-2)
**Introduction**					
Background rationale	2	Explain the scientific background and rationale for the investigation being reported	Introduction section, pages 4-5		
Objectives	3	State specific objectives, including any prespecified hypotheses	Page 5, final paragraph		
**Methods**					
Study Design	4	Present key elements of study design early in the paper	Methods section, pages 6-7		
Setting	5	Describe the setting, locations, and relevant dates, including periods of recruitment, exposure, follow-up, and data collection	Methods section, page 6, first paragraph		

For peer review only

Participants	6	*(a) Cohort study* - Give the eligibility criteria, and the sources and methods of selection of participants. Describe methods of follow-up<br>*Case-control study* - Give the eligibility criteria, and the sources and methods of case ascertainment and control selection. Give the rationale for the choice of cases and controls<br>*Cross-sectional study* - Give the eligibility criteria, and the sources and methods of selection of participants<br><br>*(b) Cohort study* - For matched studies, give matching criteria and number of exposed and unexposed<br>*Case-control study* - For matched studies, give matching criteria and the number of controls per case	Methods section, page 6, paragraphs 2 and 3	RECORD 6.1: The methods of study population selection (such as codes or algorithms used to identify subjects) should be listed in detail. If this is not possible, an explanation should be provided.<br><br>RECORD 6.2: Any validation studies of the codes or algorithms used to select the population should be referenced. If validation was conducted for this study and not published elsewhere, detailed methods and results should be provided.<br><br>RECORD 6.3: If the study involved linkage of databases, consider use of a flow diagram or other graphical display to demonstrate the data linkage process, including the number of individuals with linked data at each stage.	Methods section, page 6, paragraphs 2 and 3<br><br>N/A<br><br>N/A
Variables	7	Clearly define all outcomes, exposures, predictors, potential confounders, and effect modifiers. Give diagnostic criteria, if applicable.	Methods section, page 6, paragraph 4	RECORD 7.1: A complete list of codes and algorithms used to classify exposures, outcomes, confounders, and effect modifiers should be provided. If these cannot be reported, an explanation should be provided.	N/A
Data sources/ measurement	8	For each variable of interest, give sources of data and details of methods of assessment (measurement).<br>Describe comparability of assessment methods if there is more than one group	Methods section, page 6, paragraph 3		

For peer review only

Bias	9	Describe any efforts to address potential sources of bias	N/A		
Study size	10	Explain how the study size was arrived at	N/A		
Quantitative variables	11	Explain how quantitative variables were handled in the analyses. If applicable, describe which groupings were chosen, and why	N/A		
Statistical methods	12	(a) Describe all statistical methods, including those used to control for confounding<br>(b) Describe any methods used to examine subgroups and interactions<br>(c) Explain how missing data were addressed<br>(d) *Cohort study* - If applicable, explain how loss to follow-up was addressed<br>*Case-control study* - If applicable, explain how matching of cases and controls was addressed<br>*Cross-sectional study* - If applicable, describe analytical methods taking account of sampling strategy<br>(e) Describe any sensitivity analyses	Methods section, paragraph 3		
Data access and cleaning methods		..		RECORD 12.1: Authors should describe the extent to which the investigators had access to the database population used to create the study population.	Methods section, paragraph 3

				RECORD 12.2: Authors should provide information on the data cleaning methods used in the study.	
Linkage		..		RECORD 12.3: State whether the study included person-level, institutional-level, or other data linkage across two or more databases. The methods of linkage and methods of linkage quality evaluation should be provided.	N/A
**Results**					
Participants	13	(a) Report the numbers of individuals at each stage of the study (*e.g.*, numbers potentially eligible, examined for eligibility, confirmed eligible, included in the study, completing follow-up, and analysed)<br>(b) Give reasons for non-participation at each stage.<br>(c) Consider use of a flow diagram	Results section, page 7, first paragraph	RECORD 13.1: Describe in detail the selection of the persons included in the study (*i.e.,* study population selection) including filtering based on data quality, data availability and linkage. The selection of included persons can be described in the text and/or by means of the study flow diagram.	Methods and results section, pages 6-7
Descriptive data	14	(a) Give characteristics of study participants (*e.g.*, demographic, clinical, social) and information on exposures and potential confounders<br>(b) Indicate the number of participants with missing data for each variable of interest<br>(c) *Cohort study* - summarise follow-up time (*e.g.*, average and total amount)	Results section, page 7, first paragraph<br><br>Results section, page 7, second paragraph		
Outcome data	15	*Cohort study* - Report numbers of outcome events or summary measures over time<br>*Case-control study* - Report numbers in each exposure	Results section, page 7, second paragraph		

For peer review only

		category, or summary measures of exposure<br>*Cross-sectional study* - Report numbers of outcome events or summary measures			
Main results	16	(a) Give unadjusted estimates and, if applicable, confounder-adjusted estimates and their precision (e.g., 95% confidence interval). Make clear which confounders were adjusted for and why they were included<br>(b) Report category boundaries when continuous variables were categorized<br>(c) If relevant, consider translating estimates of relative risk into absolute risk for a meaningful time period	Results section, page 7, first paragraph in results section. Results section, third paragraph.<br><br>Tables 1-3<br><br>Figures 1-2<br><br>Results section, pages 9 - 10		
Other analyses	17	Report other analyses done—e.g., analyses of subgroups and interactions, and sensitivity analyses	N/A		
**Discussion**					
Key results	18	Summarise key results with reference to study objectives	Page 13, paragraph 1		
Limitations	19	Discuss limitations of the study, taking into account sources of potential bias or imprecision. Discuss both direction and magnitude of any potential bias	Page 13, paragraph 3 and page 16, paragraph 2	RECORD 19.1: Discuss the implications of using data that were not created or collected to answer the specific research question(s). Include discussion of misclassification bias, unmeasured confounding, missing data, and changing eligibility over time, as they pertain to the study being reported.	N/A
Interpretation	20	Give a cautious overall interpretation of results considering objectives,	Discussion section, pages 13-16		

		limitations, multiplicity of analyses, results from similar studies, and other relevant evidence			
Generalisability	21	Discuss the generalisability (external validity) of the study results	Page 16, final paragraph of discussion		
**Other Information**					
Funding	22	Give the source of funding and the role of the funders for the present study and, if applicable, for the original study on which the present article is based	Page 16		
Accessibility of protocol, raw data, and programming code		..		RECORD 22.1: Authors should provide information on how to access any supplemental information such as the study protocol, raw data, or programming code.	Data sharing statement provided page 20

*Reference: Benchimol EI, Smeeth L, Guttmann A, Harron K, Moher D, Petersen I, Sørensen HT, von Elm E, Langan SM, the RECORD Working Committee. The REporting of studies Conducted using Observational Routinely-collected health Data (RECORD) Statement. *PLoS Medicine* 2015; in press.

*Checklist is protected under Creative Commons Attribution (CC BY) license.

For peer review only
